# Enhancing grasshopper optimization algorithm (GOA) with levy flight for engineering applications

**DOI:** 10.1038/s41598-022-27144-4

**Published:** 2023-01-04

**Authors:** Lei Wu, Jiawei Wu, Tengbin Wang

**Affiliations:** 1 Information College, North China University of Technology, Beijing, 100144 China; 2 Faculty of Architecture, Civil and Transportation Engineering, Beijing University of Technology, Beijing, 100124 China

**Keywords:** Civil engineering, Mechanical engineering

## Abstract

The grasshopper optimization algorithm (GOA) is a meta-heuristic algorithm proposed in 2017 mimics the biological behavior of grasshopper swarms seeking food sources in nature for solving optimization problems. Nonetheless, some shortcomings exist in the origin GOA, and GOA global search ability is more or less insufficient and precision also needs to be further improved. Although there are many different GOA variants in the literature, the problem of inefficient and rough precision has still emerged in GOA variants. Aiming at these deficiencies, this paper develops an improved version of GOA with Levy Flight mechanism called LFGOA to alleviate the shortcomings of the origin GOA. The LFGOA algorithm achieved a more suitable balance between exploitation and exploration during searching for the most promising region. The performance of LFGOA is tested using 23 mathematical benchmark functions in comparison with the eight well-known meta-heuristic algorithms and seven real-world engineering problems. The statistical analysis and experimental results show the efficiency of LFGOA. According to obtained results, it is possible to say that the LFGOA algorithm can be a potential alternative in the solution of meta-heuristic optimization problems as it has high exploration and exploitation capabilities.

## Introduction

Till date, researchers and practitioners have presented and experimented with various nature-inspired metaheuristic algorithms to handle various search problems. O. N. Oyelade et al.^1^ (2022) proposed an appealing Ebola Optimization Search Algorithm, they achieved some attractive results, especially when the EOSA algorithm was applied to address the complex problem of selecting the best combination of convolutional neural network (CNN) hyperparameters in the image classification of digital mammography. But the mathematical model of EOSA is a little complicated. Laith Abualigah et al.^2^ (2022) proposed a unique Reptile Search Algorithm (RSA) and achieved better results than the other competitive optimization algorithms when applied their RSA algorithm to solve seven real-world engineering problems. Since the RSA algorithm introduction, many RSA variants have been proposed. It will be better; if they gave the statistical numerical results (such as mean and standard deviation) of the RSA algorithm and other comparative algorithms in solving seven engineering problems. Abualigah, Laith Mohammad et al.^3^ (2021) proposed a novel mathematically modelled: Arithmetic Optimization Algorithm (AOA), that utilizes the main arithmetic operators: Multiplication (M), Division (D), Subtraction (S), and Addition (A). Although, the better performance of the AOA is evaluated using twenty-nine benchmark functions and several real-world engineering design problems. But the parameter Math Optimizer Accelerated (MOA) is increased linearly from 0.2 to 0.9 still needs extensively discussed. Hussien, A.G et al.^4^ (2022) comprehensively reviewed the recent widespread applications and variants of Harris hawk optimizer (HHO) in-depth. The authors thoughtfully investigated several possible future directions and possible ideas of the recent applications and variants of well-established HHO. As soon as Snake Optimizer (SO) is proposed (2022) by Fatma A. Hashim et al.^5^, the SO algorithm attracted researchers and practitioners and the SO algorithm was applied to many dominions, as the SO optimization algorithm is simple and efficient. Since the SO algorithm introduction, many SO variants have been proposed to tackle optimization problems. Zheng, Rong et al.^6^ (2022) proposed an improved wild horse optimizer (IWHO) integrated three improvements: random running strategy (RRS), dynamic inertia weight strategy (DIWS), and competition for waterhole mechanism (CWHM). The IWHO algorithm has successfully overcome the crucial drawbacks of the origin WHO may be stuck in local optimal regions or has a slow convergence. The IWHO algorithm is evaluated by classical benchmark functions and five real-world optimization problems and compared with nine well-known algorithms. Huangjing Yu et al.^7^ (2022) proposed an improved Aquila optimizer (mAO), the highlight of the mAO algorithm is the restart strategy, which is simple but effective. Their mAO algorithm has solved five engineering optimization problems; but has not been compared with other algorithms by numerical statistics such as mean and standard deviation. Feature selection problem is one of the main difficulties in machine learning domain to find the smaller number of informative features among a huge amount of feature space which guides the maximum classification ratio. Hussien, A.G., Amin, M.^8^ (2022) proposed an improved version of HHO called IHHO, which not only solves 5 constrained engineering problems but also has been applied to solve feature selection problems using 7 UCI datasets.

Pengchuan Wang et al.^9^ (2020) comprehensively and extensively overviewed the recent widespread applications and variants of Complex-valued encoding algorithm in-depth. The authors successfully tested eight complex-valued encoding algorithms by standard benchmark functions and solved five engineering optimization design problems. But the mathematical model of Complex-valued encoding algorithm is a little complicated. Chen et al^10^ (2020) proposed an improved arithmetic optimization algorithm (IAOA) based on the population control strategy to solve numerical optimization problems, which successfully solved optimization problems to consume less energy during robotic arm movement.

### Grasshopper optimisation algorithm, variants, and applications

According to the behavior of grasshopper swarms in nature, Shahrzad Saremia. et al. in 2017 proposed a unique and novel swarm intelligence algorithm called the grasshopper optimization algorithm (GOA)^11^, making utilization of the swarm intelligence to solve optimization problems. This algorithm is proven to be efficient in solving global unconstrained and constrained optimization problems. Since 2017, GOA has attracted increasing interest from academics and researchers, most researchers and practitioners have achieved success with the GOA algorithm to solve various complex and real-world problems in many different domains^12–14^. On the other hand, to fully extend the performances of the GOA, most researchers and practitioners constructed a variety of hybrid variants^15^ based on GOA and other metaheuristics; and embedded different key parameters into the GOA, to solve their practical fields’ complex real-world problems. Arora, S et al.^16^ (2019) introduced the chaotic method with GOA for solving global optimization. Zhao, S et al.^17^ (2021) embedded trigonometric substitution into GOA to enhance Cauchy mutation. Ahmed A et al.^18^ (2022) merged Crossover Operators with GOA for feature selection and solving engineering problems. Yildiz et al.^19^ (2021) proposed using elite opposition-based learning to enhance GOA for solving real-world engineering problems. Yi Feng et al.^20^ (2020) introduced Dynamic Opposite Learning assisted GOA for the Flexible Job Scheduling Problem. Qin, P et al.^21^ (2021) have successfully applied improved GOA to optimise the parameters of the BP neural network for predicting the closing prices of the Shanghai Stock Exchange Index and the air quality index (AQI) of Taiyuan, Shanxi Province.

### The nature-inspired meta-heuristic algorithm with levy flight

Wang, Shuang et al.^22^ (2022) proposed an improved version of ROA called Enhanced ROA (EROA) using three different techniques: adaptive dynamic probability, SFO with Levy flight, and restart strategy; and have successfully overcome slow convergence and stagnation in local optima of the origin ROA. As soon as the Levy flight trajectory-based WOA (LWOA) algorithm is proposed by Zhou, Y., Ling, Y. and Luo, Q.^23^ (2018), which attracted researchers and practitioners and applied the LWOA algorithm to many dominions, because the LWOA algorithm effectively adaptation, few control parameters, and simplicity of structure. Xuan Chen et al.^24^ (2021) employments of Opposition-based learning and the Genetic algorithm with Levy’s flight to improve the Wolf Pack Algorithm and achieved maintain the diversity of the initial population during the global search. Their experimental results show that their proposed algorithm has a better global and local search capability, especially in the presence of multi-peak and high-dimensional functions.

Above mentioned cases are only a few typical models, but they show the nature-inspired meta-heuristic algorithm gets the best global value largely dependent on together with levy flight. On the other hand, these studies affirm that levy flight can considerably enhance the performance of meta-heuristic optimizers.

Our main contribution is to use the grasshopper optimization algorithm with Levy Flight distribution strategy (LFGOA) to seven real-world problems, which cover hybrids (continuous, discrete, and integer variables) nonlinear constrained optimization, such as Himmelblau’s nonlinear optimization problem, Cantilever beam design, Car Side Impact Design, Gear train Design, Pressure vessel design, Speed Reducer Design, and tabular column design.

Another contribution is that the levy flight strategy is properly embedded with GOA to help explore the search space. The comprehensive effect of levy flight mechanisms strengthens the exploration-exploitation balance during the search process.

The third contribution is that performance of the LFGOA algorithm was validated by 23 mathematical benchmark functions in comparison with the eight well-known meta-heuristic algorithms (AHA, AO, DA, DMOA, GBO, HGS, HHO, and MVO) and the comprehensive performance of the LFGOA algorithm is superior to the eight algorithms and the origin GOA algorithm.

The fourth contribution is that the extensibility test with different scales of dimensions 50, 100, 300, and 500, is undertaken by comparing LFGOA with the original GOA to assess the dimensional influence on problem consistency and optimization quality. The comparisons show that the proposed LFGOA algorithm still holds a simple and efficient structure that significantly improves the performance of the origin GOA algorithm.

In the rest of this paper, Section 2 provides the key idea and structure of the Grasshopper Optimization Algorithm (GOA). Section 3 provides Grasshopper Optimization Algorithm with Levy Flight (LFGOA), improvement steps in in-depth and LFGOA pseudo-code. Section 4 extensively introduces the experimental design and simulation results. Section 5 presents seven real applications of LFGOA in nonlinearly-constrained engineering optimization problems. Finally, section 6 concludes the paper and future directions.

## The grasshopper optimization algorithm (GOA)

**Figure 1:**
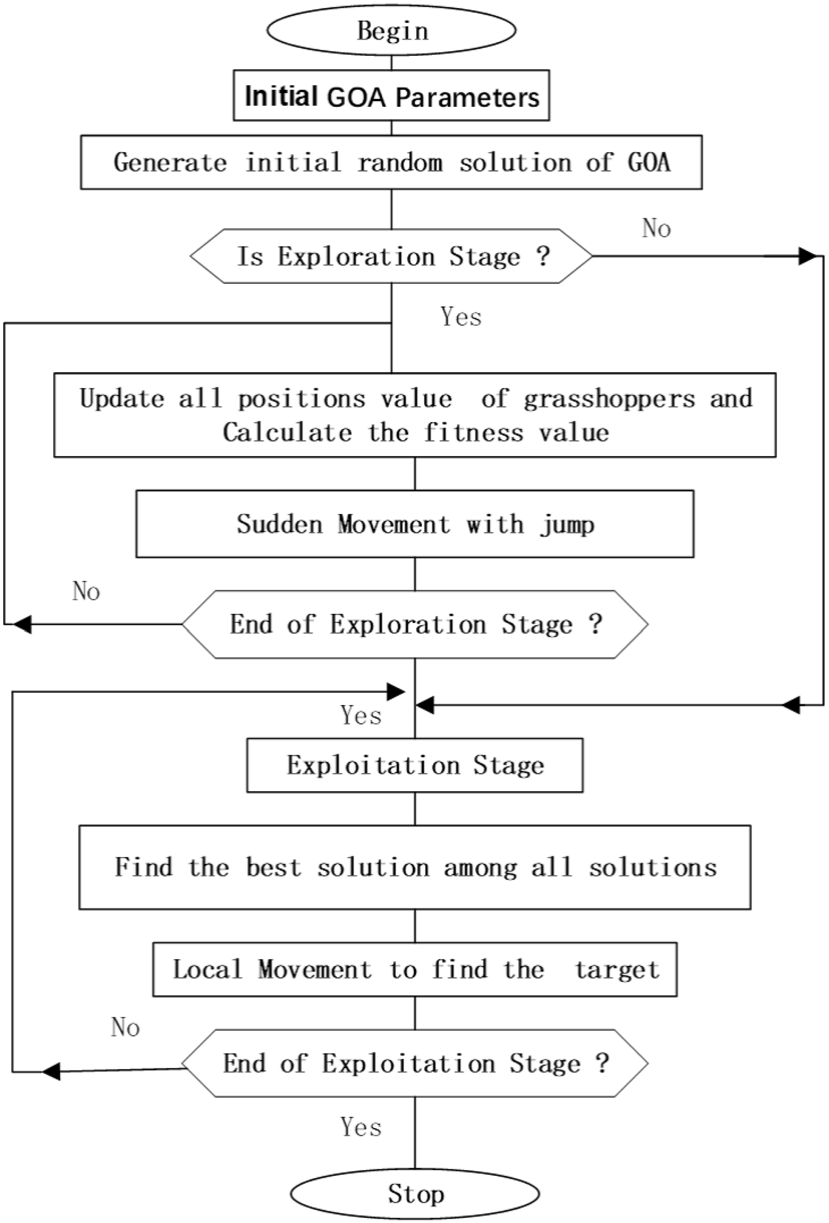
The grasshopper swarm searches with two stages.

GOA algorithm is inspired by the foraging and swarming behavior of grasshoppers in nature for solving numerical optimization issues. The life cycle of the grasshopper includes two stages called nymph and adulthood. The nymph stage is characterized by small steps and slow movements, while the adulthood stage is characterized by long-range and abrupt movements. The movements of nymphs and adulthood constitute the intensification and diversification phases of GOA. Intuitively speaking, the GOA search process splits into two stages: exploration and exploitation are shown in Fig. 1.

In the exploration stage, we update all the positions’ values and compute the fitness value of all grasshopper swarms (search for food sources). In the exploitation stage, we find the best solution among all solutions (search for better food sources).

### Principal of the grasshopper optimization algorithm

In the GOA algorithm, each grasshopper represents a solution in the population. The grasshopper swarms behavior is mathematically modelled and used to calculate the position $X_i$ of each solution as follows:

1 $$\begin{aligned} X_i=S_i+G_i+A_i \end{aligned}$$

where $X_i$ indicates the ith grasshopper’s position, $S_i$ denotes the grasshopper interaction between the solution and the other grasshoppers’ swarms, $G_i$ is the gravity force on the ith solution, and $A_i$ represents the wind advection, which can be represented by the below equations:

2 $$\begin{aligned} S_i= & {} \sum _{j=1}^{N}s\left( d_{ij}\right) \widehat{{\ d}_{ij}},\ \ \ \ \ \ \ \ \ \ \ \ \ \ \ \ \ \ where\ i\ne j \end{aligned}$$

3 $$\begin{aligned} s= & {} fe^\frac{-r}{l}-e^{-r} \end{aligned}$$

where *N* denotes the number of grasshoppers, $d_{ij}= |x_j-x_i|$ defines the Euclidean distance between the *ith* and the *jth* grasshoppers swarm, $\widehat{d_{ij}}=\frac{| x_j-x_i|}{d_{ij}}$ represents the unit vector from the *ith* to the *jth* grasshopper swarm. In addition, *s* represents the strength of two social forces (repulsion and attraction between grasshopper swarms), where *l* is the attractive length scale and *f* is the intensity of attraction.

When the distance between two grasshoppers swarm in the range [0, 2.079], repulsion occurs, and when the distance between two grasshoppers swarm is exactly 2.079, neither attraction nor repulsion occurs, which forms a comfort zone. When the distance exceeds 2.079, the attraction force increases, then progressively decreases until it reaches 4. The function *s* fails to apply forces between grasshoppers’ swarms when the distance between them is larger than 10. To solve this problem, we map the distance of grasshoppers’ swarms in the interval [1, 4].

The equation below shows how to calculate the force of gravity $G_i$:

4 $$\begin{aligned} G_i=-g\widehat{e_g} \end{aligned}$$

where *g* denotes the gravitational constant and $\widehat{e_g}$ is unit vector toward center of earth.

The equation below shows how to compute $A_i$:

5 $$\begin{aligned} A_i=u\widehat{e_w} \end{aligned}$$

where *u* represents the drift constant and $\widehat{e_w}$ is the unit vector in the wind direction.

After replacing the values of $S_i$, $G_i$, and $A_i$, equation (1) can be reconstructed as follows by Equations 2, 3, 4 and 5:

6 $$\begin{aligned} \begin{aligned} X_i=&\sum _{j=1}^{N}{s\left( d_{ij}\right) \widehat{d_{ij}}}-g\widehat{e_g}+u\widehat{e_w} \\ =&\sum _{j=1}^{N}{s\left( | x_j-x_i| \right) \widehat{\frac{| x_j-x_i|}{d_{ij}}}}-g\widehat{e_g}+ u\widehat{e_w} \ \ \ \ \ \ \ \ \ \ where \ \ i\ne j \end{aligned} \end{aligned}$$

However, the mathematical model of equation (6) cannot be used directly to solve the optimization problems, as mainly the grasshoppers quickly reach their comfort zone and the grasshopper’s swarms from failing to converge to the location target or a specified point (global optimum). To solve optimization issues and prevent grasshopper swarms from quickly reaching their comfort zone, the equation truly actuarily applied to solve optimization problems is proposed by the author as follows:

7 $$\begin{aligned} X_i^d=c\left( \sum _{j=1}^{N}{c\frac{UB_d-LB_d}{2}}s(| x_j^d-x_i^d|)\frac{| x_j-x_i|}{d_{ij}}\right) +\widehat{T_d} \end{aligned}$$

where $UB_d$ and $LB_d$ are the upper and lower bounds in the dth dimension respectively, $\widehat{T_d}$ denotes the best solution found so far in the dth dimension space. In Eq. (7), the gravity force is not considered, that is, there is no $G_i$ component. And assume that the wind direction ($A_i$ component) is always towards a target $T_d$. The second term $\widehat{T_d}$, simulates the tendency of grasshoppers to move towards the food source.

### The key parameter *c* in mathematical model

In the grasshopper swarm algorithm, parameter *c* in Eq. (7) is very important for local and global search. The inner *c* in Eq. (7) is used to reduce the repulsion, attraction and comfort zone between grasshoppers correspondingly to the number of iterations; is also responsible for the reduction of repulsion/attraction forces between grasshoppers’ swarms, which is proportional to the number of iterations. The outer *c* in Eq. (7) is responsible to reduce the grasshopper’s movements around the target (food) and helps reduce the search coverage around the target as the iteration goes on increasing. The coefficient *c* is proposed as follows:

8 $$\begin{aligned} c=c_{max}-t\frac{c_{max}-c_{min}}{t_{max}} \end{aligned}$$

where $c_{max}$ and $c_{min}$ are the maximum and minimum values of *c* respectively, $c_{max}$ and $c_{min}$ can be set as 1 and 0.00001 respectively, where *t* is the current iteration, and $t_{max}$ is the maximum iteration value. The position of a grasshopper is updated based on its current position, the global best position, and the positions of other grasshoppers within the swarm.

## The grasshopper optimization algorithm with levy flight

### Mantegna’s algorithm from levy flights random walks

The study shows that the distribution probability density function of the variation of the Levy’s flight step can be approximated as follows:

9 $$\begin{aligned} L(s) \sim |s|^{- 1 - \theta }. \end{aligned}$$

Where *s* is the random step length of Levy’s flight behavior, and $\theta$ is bounded as [0, 2] as a power-law index and is set to be 1.5, which controls the peak sharpness of the levy distribution graph. The different values of the parameter $\theta$ cause different distributions, it makes longer jumps for smaller values, whereas it makes shorter jumps for bigger values. True Levy distribution is hard to implement in computer code, but the approximate form. Mantegna algorithm is one of the fast and accurate algorithms which generate a stochastic variable whose probability density is close to the Levy stable distribution characterized. Mantegna’s algorithm can be split into three steps. For random walks, Mantegna’s algorithm determines the step length *S* as follows:

10 $$\begin{aligned} S=\frac{U}{|V|^\frac{1}{\theta }}, \end{aligned}$$

where *S* is the random step length variable, while *U* and *V* are two normal stochastic variables with standard deviation $\sigma _U$ and $\sigma _V$, *U* and *V* should be attained based on normal distributions:

11 $$\begin{aligned} U \sim N\left( {0,~\sigma _{U}^{2}} \right) ,~V \sim \left( {0,\sigma _{V}^{2}} \right) \end{aligned}$$

The symbol $\sim$ in Eq. (11) denotes the random variable obeys the distribution on the right-hand side; that is, samples should be drawn from the distribution. As the standard deviation $\sigma _U$ and $\sigma _V$ cannot be chosen independently for an arbitrary value of $\theta$, for simplicity we usually set

12 $$\begin{aligned} \sigma _V=1 \end{aligned}$$

After this setting, the standard deviation $\sigma _U$ can be obtained by:

13 $$\begin{aligned} \sigma _{U} = \left\{ \frac{\Gamma (1 + \theta ) \times sin(0.5\pi \theta )}{\Gamma \left[0.5\left( {1 + \theta } \right) \right]\times \theta \times 2^{0.5(\theta - 1)} } \right\} ^{\frac{1}{\theta }} \end{aligned}$$

The step size of Levy flight has been achieved by the Eqs. (9) – (13), which simulates the search of short walking distance and occasionally longer walking distance. Then the step size is calculated by

14 $$\begin{aligned} step\quad size = f\times S \end{aligned}$$

Where, the factor value $f(f=0.01)$ derived from *L*/100 determines the levy walks and the factor is dependent on the dimension of the desired problem, where *L* is the wide-scale; unless Levy flights become too aggressive, it helps the new solution move away from the search space. The process of Levy flight can be exhibited in Algorithm 1.

**Figure:**
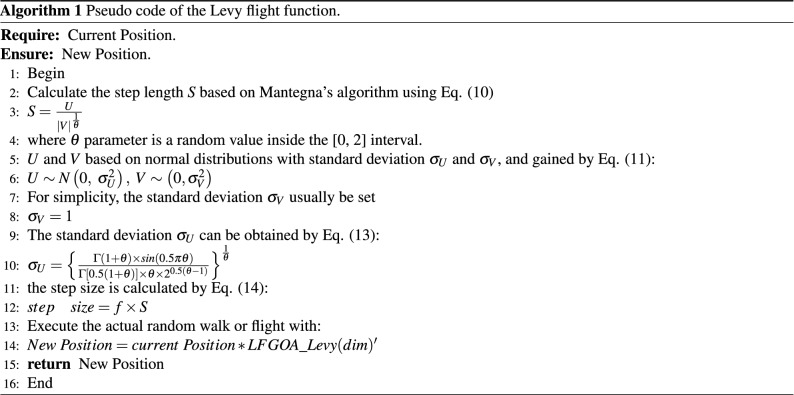

The step size value will be added to update the equations of the LFGOA algorithm for finding the best position. From theoretical perspectives, this random walk is based on a long tail distribution which can be used to help an algorithm escape from getting stuck at a local optimum^25–27^. In other words, the Levy flight distribution is an effective mathematical operator for producing varied solutions in the searching space and increasing the exploration capability of the LFGOA algorithm.

From Algorithm 1, it is worth noting the formula:

$$New Position = current Position * LFGOA\_Levy(dim)';$$

Firstly, $LFGOA\_Levy(dim)$ represents the Levy flight function, and *dim* is the dimension size of the problem. the Levy flight Strategy is integrated into the GOA by the above formula. The Levy flight has a relatively high probability of large strides in random walking, which can effectively improve the randomness of the GOA algorithm. This way, the risk that the algorithm gets stuck in a local optimum is drastically reduced, while it is still possible to perform sufficient local refinements. In other words, the algorithm presents a natural balance between exploration and exploitation.

Secondly, in the case of stagnation, Levy-triggered searching (hunting) patterns can help LFGOA to jump out of them toward new better positions. By this mechanism, the LFGOA algorithm can overcome the deficiencies of the little diversity of the origin GOA algorithm and greatly increase the probability of getting the best position (solution), which is also the highlight and unique feature of the LFGOA algorithm.

Despite being a simple change in the LFGOA algorithm, this new distribution induces drastic changes in the optimization process, LFGOA-based jumps can redistribute grasshoppers around the fitness landscape to prevent the population from the loss of diversity and to put more emphasis on the global searching tendency.

### Enhancing grasshopper optimization algorithm (GOA) with levy flight

How and where place Levy Flight in the GOA algorithm will directly produce totally different results, in some cases even give worse results. Based on the above facts, through an in-depth comprehensive study and trial-and-error experiments, we successfully embedded Levy flight into the GOA algorithm by the following simple but effetely mechanisms.

**Figure 2:**
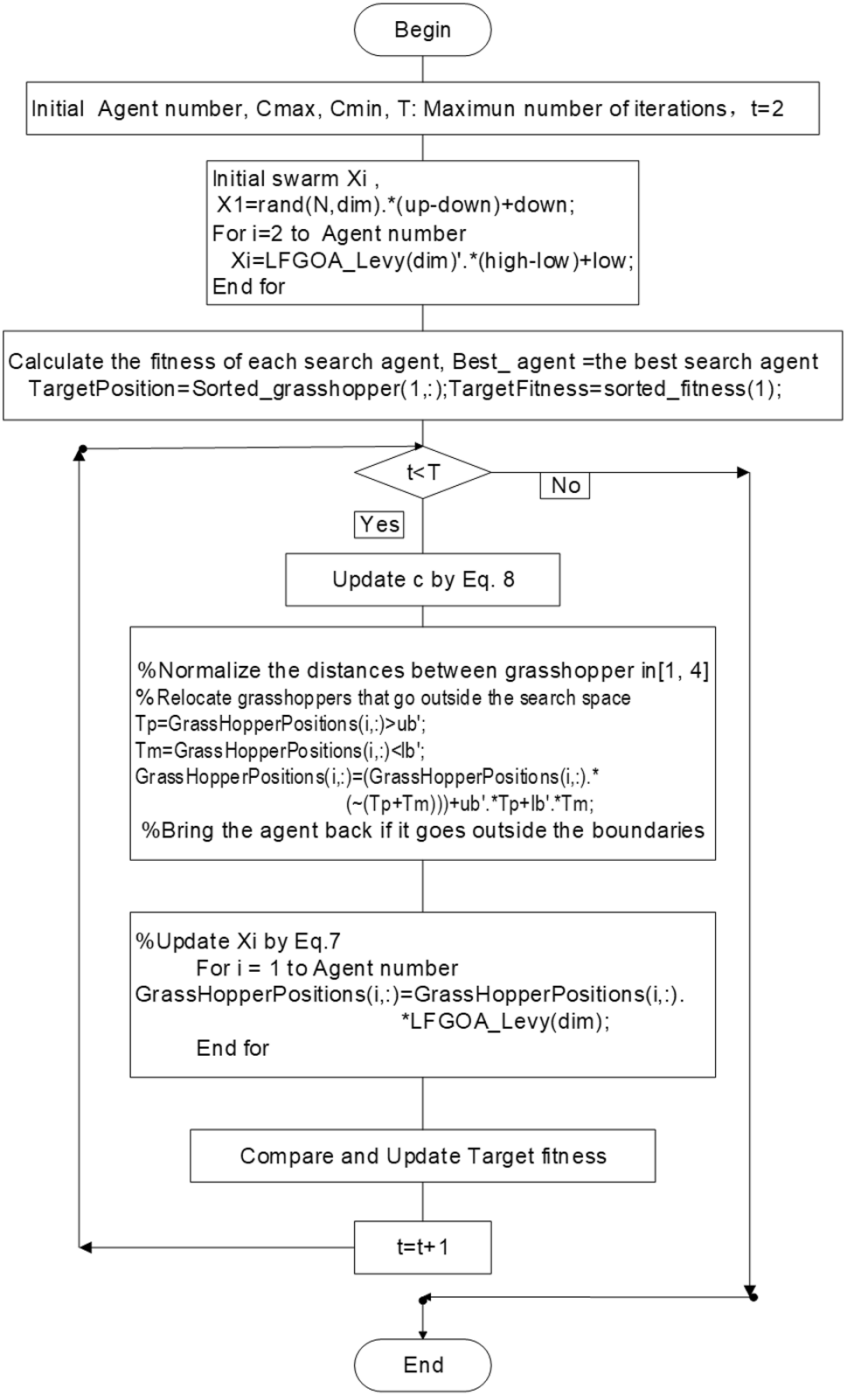
The flow chart of the LFGOA algorithm.

Firstly, except for the first grasshopper initialled with rand values (since the first iteration was dedicated to calculating the fitness of the grasshopper), the other grasshoppers were assigned Levy flight distribution values, not rand values, which directly produced a better start for most of the grasshoppers with wide diversity in the initialization stage. Secondly, the target is achieved by the Levy flight mechanism during executing iteration, which overcomes the deficiencies and can be escaped from a local optimum and restarted in a different region of the search space for the LFGOA. The flow chart of the Levy flight mechanism embedded in the GOA is shown in Fig. 2. The pseudo-code of the LFGOA algorithm is presented in Algorithm 2.

**Figure:**
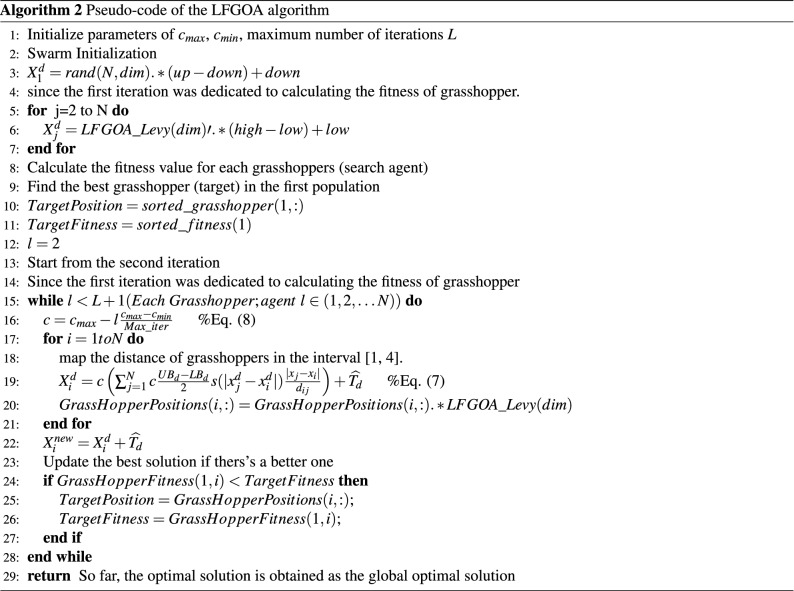


In sharp contrast: although the existing method has greatly improved GOA, there is still a large probability of falling into local optimum by the reason of immature convergence, and the truth reason derived from the diversity is underdeveloped for the GOA algorithm. On the other hand, initializes the position of agents in the search space by Levy flight as the below formula:

$$\begin{aligned} X_i=LFGOA\_Levy(dim)\prime .*(high-low)+low. \end{aligned}$$

The above formula, $LFGOA\_Levy (dim)$ represents the Levy flight function, and *dim* is the dimension size of the problem, which provides a large-scale deployment schema for the LFGOA algorithm, all grasshoppers assigned Levy flight value not random numbers between [0, 1] from the uniform distribution at the initialization stage, which directly increase the wide diversity of the LFGOA algorithm. Secondly, randomization is more efficient as the step length is heavy-tailed random redistribution, and any large step is possible, which effectively increases the probability of LFGOA’s global search ability and precision.

From Fig. 2, it is worth noting the following three formulas:

$$\begin{aligned}{} & {} Tp=GrassHopperPositions(i,:)>ub';\\{} & {} Tm=GrassHopperPositions(i,:)<lb';\\{} & {} GrassHopperPositions(i,:)=(GrassHopperPositions(i,:).*(\sim(Tp+Tm)))+ub'.*Tp+lb'.*Tm. \end{aligned}$$

Where Tp is assigned logical ‘0’. when the value of the grasshoppers’ position is less than the upper boundary, otherwise Tp is assigned logical ‘1’.

Where Tm is assigned logical ‘0’. when the value of the grasshoppers’ position is more than the lower boundary, otherwise Tm is assigned logical ‘1’.

Where $( \sim (Tp+Tm))$ is assigned to value 1 when the grasshoppers’ position is not at the boundary, otherwise is assigned to value 0.

When the grasshoppers go outside the search space, the grasshoppers will be drawn back by the above formula. After that, the positions of the grasshoppers are directly replaced (similar restarted)^28^ by the below formula:

$$\begin{aligned} GrassHopperPositions(i,:)=GrassHopperPositions(i,:).*LFGOA\_Levy(dim). \end{aligned}$$

Based on the above formula, the positions of all the grasshoppers random redistribution around the fitness landscape to prevent the population from the loss of diversity and to put more emphasis on the global searching tendency. The balance between exploration and exploitation can be achieved according to the Levy flight based jumps, which allows grasshoppers to escape from local minima and explore different search areas. However, it cannot ensure the new update position is better than the current position.

### The proposed approach

As a newly proposed algorithm, GOA has achieved good results on some test functions. However, experiment results show that it still has the defects of insufficient global exploration and local optimum stagnation. The lack of global exploration capacity can be attributed to the deficient searches with two stages. Thus, GOA properly integrated with Levy Flight is utilized to improve the global search ability in this work. Meanwhile, a restart strategy of Levy Flight is added to GOA that helps the GOA algorithm escape from local optima.

To the best of our knowledge, the main reason behind the effectiveness of LFGOA is that the Levy flight based jumps can effectively redistribute the search agents to enhance their diversity and to emphasize more explorative steps in case of immature convergence to local optima. It is a successful GOA variant of combining GOA with Levy Flight and gained better results of applying LFGOA in seven real-world engineering problems. The statistical analysis and experimental results show the efficiency of LFGOA.

In section 4, the strict experiments will exhibit that LFGOA is superior to the GOA algorithm in most performance metrics, especially at the parts of correct getting the best solutions with quick convergence speed. In fact, LFGOA still holds the advantages of simple structure and few-parameter-turnings even added extra Levy flight mechanism.

## Experimental results and analysis

In this section, all experiments were carried out under the Windows 10 OSx64 using MATLAB R2019a software, and the hardware platform used was configured with Intel(R) Core (TM) i7-8700 CPU @ 3.20GHz and 8 GB RAM.

The performance of the suggested LFGOA is assessed in this section by using five experiments. Accordingly, the first one evaluates AHA, AO, DA, DMOA, GBO, HGS, HHO, LFGOA, and MVO about the average value, the standard deviation, and the best value using twenty-three mathematical benchmark functions presented in Table 1. These benchmark functions are categorized into three groups: unimodal, multi-modal, and composite.

Here, the LFGOA performance is tested using twenty-three benchmark functions. This benchmark contains seven unimodal, six multimodal, and ten fixed-dimension multimodal functions. The mathematical description of each type is given in Table 1 where *N* denotes the number of grasshoppers, *T* refers to the maximum iteration value, dim refers to the number of dimensions, Range shows the interval of search space, $F_{min}$ refers to the optimal value that the corresponding functions can achieve.

The second one strictly tests the convergence performance of the LFGOA with AHA, AO, DA, DMOA, GBO, HGS, HHO, and MVO respectively. The third experiment aims to test the LFGOA by a non-parametric Wilcoxon, Friedman, and Nemenyi statistical test. The fourth tests the scalability performance of the LFGOA compared with the GOA comprehensively and thoroughly under conditions of 50, 100, 300, and 500 Dimensions. The fifth part presents some quantitative metrics of LFGOA.

**Table 1:** Twenty-three benchmark functions.

Function	Dimensions	Range	$F_{min}$
	(N, T, dim)		
**Univariate test functions**			
F1:sphere function	100,100,30	[−100, 100]	0
$$F_1\left( x\right) =\sum _{i=1}^{dim}x_i^2$$			
F2: Schwefel’s problem 2.22	100,100,30	[−100, 100]	0
$$F_2(x)=\sum _{i=1}^{dim}{|x_i|}+\prod _{i=1}^{dim}{|x_i|}$$			
F3: Shifted schwefel’s problem 1.2	100,100,30	[−10, 10]	0
$$F_3(x)=\sum _{i=1}^{dim}\sum _{j=1}^{i}x_j^2$$			
F4: Schwefel’s problem 2.21	100,100,30	[−100, 100]	0
$$F_4(x)={max}_i{|x_i|,1\le i\le dim}$$			
F5: Generalized rosenbrock’s function	100,100,30	[−100, 100]	0
$$F_5(x)=\sum _{i=1}^{dim-1}{[100\left( x_{i-1}-x_i^2\right) +{(x_i-1)}^2}$$			
F6: Step function	100,100,30	[−30, 30]	0
$$F_6(x)=\sum _{i=1}^{dim}{(|x_i+0.5|)}^2$$			
F7:Quartic function i.e. noise	100,100,30	[−1.28, 1.28]	0
$$F_7(x)=\sum _{i=1}^{dim}{iX_i}^4+random[0,1]$$			
**Multidimensional test functions**			
F8: Generalized schwefel’s problem 2.26	100,100,30	[−500, 500]	$-418.9829*dim$
$$F_8\left( x\right) =\sum _{i=1}^{dim}\left| x_i\right| -x_i\sin (\sqrt{|x_i|})$$			
F9: Generalized rastrigin’s function	100,100,30	[−5.12, 5.12]	0
$$F_9(x)=\sum _{i=1}^{dim}\left[ x_i^2-10\cos {\left( 2\pi x_i\right) }+10\right]$$			
F10: Ackley’s function	100,100,30	[−32, 32]	0
$$F_{10}\left( x\right) =-20\exp {\left( -0.2\sqrt{\frac{1}{dim}}\sum _{i=1}^{dim}x_i^2\right) }-\exp {\left( \frac{1}{dim}\sum _{i=1}^{dim}cos\left( 2\pi x_i\right) \right) }+20+e$$			
F11: Generalized griewank’s function	100,100,30	[−600, 600]	0
$$F_{11}\left( x\right) =\frac{1}{4000}\sum _{i=1}^{dim}x_i^2-\prod _{i=1}^{dim}{\cos {\left( \frac{x_i}{\sqrt{i}}\right) }+1}$$			
F12: Generalized penalized function 1	100,100,30	[−50, 50]	0
$$F_{12}(x)=\frac{\pi }{dim}10{sin}^2(\pi y_1)+\sum _{i=1}^{n-1} (y_i-1)^2[1+\sin ^2(\pi y_{i+1)}]+(y_{dim}-1)^2+\sum _{i=1}^{n}Ufun(xi,10,100,4)$$			
F13: Generalized penalized function 2	100,100,30	[−50, 50]	0
$$F_{13}\left( x\right) =0.1\left\{ \sin ^2(3\pi x_1)+\sum _{i=1}^{dim}{(x_i-1)}^2[1+\sin ^2(3\pi x_{i+1})]+{(x_{dim}-1)}^2[1+\sin ^2(2\pi x_{dim})]+\sum _{i=1}^{dim}Ufun(x_i,5,100,4) \right\}$$			
where $$Ufun\left( x_i,5,100,4\right) =\left\{ \begin{matrix}k{(x_i-a)}^m\ \ \ \ \ \ \ \ x_i>a\\ 0-a\ \ \ \ \ -a<x_i<a\\ k{(-x_i-a)}^m\ \ \ x_i<-a\\ \end{matrix}\right.$$			
$$y_i=1+\frac{x_i+1}{4}$$			
**Composite multidimensional (or fixed multidimensional ) test functions**			

Function	Dimensions	Range	$F_{min}$
	(N, T, dim)		
F14: Shekel’s foxholes function	100,100,2	[−65.5360, 65.5360]	0.9980
$$F_{14}\left( x_1,x_2\right) ={(\frac{1}{500}+\sum _{j=1}^{25}\frac{1}{j+\sum _{i=1}^{2}{(x_i-a_{ij})}^6})}^{-1}$$			
$$a_{1j}= \left\{ -32;-16; 0; 16; 32; -32; -16; 0; 16; 32; -32;-16; 0; 16; 32; -32; -16; 0; 16; 32; -32; -16; 0; 16; 32\right\}$$			
$$a_{2j} = \left\{ -32;-32; -32;-32;-32;-16;-16;-16;-16;-16; 0; 0; 0; 0; 0; 16; 16; 16; 16; 16; 32; 32; 32; 32; 32\right\}$$			
F15: Kowalik’s function	100,100,4	[−5, 5]	0.0003075
$$F_{15}\left( x\right) =\sum _{k=1}^{11}[a_k-\frac{x_1(b_k^2+b_kx_2)}{b_k^2+b_kx_3+x_4}]^2$$			
$$\begin{matrix} i &{} a_i &{} b_i^{-1}\\ 1 &{} 0.1957 &{} 0.25\\ 2 &{} 0.1947 &{} 0.5\\ 3 &{} 0.1735 &{} 1\\ 4 &{} 0.16 &{} 2\\ 5 &{}0.0844 &{} 4\\ 6 &{} 0.0627 &{} 6\\ 7 &{} 0.0456 &{} 8\\ 8 &{} 0.0342 &{} 10\\ 9 &{} 0.0323 &{} 12\\ 10 &{} 0.0235 &{} 14\\ 11 &{} 0.0246 &{} 16 \\ \end{matrix}$$			
F16: Six-hump camel-back function	100,100,2	[−5, 5]	−1.0316285
$$F_{16}\left( x_1,x_2\right) =4x_1^2-2.1x_1^4+\frac{1}{3}x_1^6+x_1x_2-4x_2^2+4x_2^4$$			
F17: Branin function	100,100,2	[−5, 0, 10, 15]	0.397887
$$F_{17}\left( x_1,x_2\right) =(x_2-\frac{5.1}{4{\pi }^2}x_1^2+\frac{5}{\pi }x_1-6)^2+10\left( 1-\frac{1}{8\pi }\right) cosx_1+10$$			
F18: Goldstein-price functionn	100,100,2	[−5, 5]	3
$$F_{18}\left( x_1,x_2\right) =[1+(x_1+x_2+1)^2(19-14x_1+3x_1^2-14x_2+$$			
$$6x_1x_2+3x_2^2)]\times [30+(2x_1-3x_2)^2-3x_2)^2$$			
$$\times \left( 18-32x_1+12x_1^2+48x_2-36x_1x_2+27x_2^2\right) ]$$			
**F19 and F20:Hartman’s Family**			
F19: Hartman’s family	100,100,3	[0,1]	-3.863
$$F_{19}\left( x\right) =-\sum _{i=1}^{4}{c_i\exp (-\sum _{j=1}^{3}{a_{ij}(x_j-p_{ij})^2)}}$$			
F20: Hartman’s family	100,100,6	[0,1]	−3.322
$$F_{20}\left( x\right) =-\sum _{i=1}^{4}{c_i\exp (-\sum _{j=1}^{6}{a_{ij}(x_j-p_{ij})^2)}}$$			
Where, in this exercise: $$\begin{matrix} i &{} &{} a_{ij} &{} &{} c_{i} &{} &{} p_{ij} &{} \\ 1 &{} 3.0 &{} 10.0 &{} 30.0 &{} 1.0 &{} 0.689 &{}0.1170 &{} 0.2673\\ 2 &{} 0.1 &{} 10.0 &{} 35.0 &{} 1.2 &{} 0.4699 &{} 0.4387 &{} 0.7470\\ 3 &{} 3.0 &{} 10.0 &{} 30.0 &{} 3.0&{} 0.1091&{} 0.8732 &{} 0.5547\\ 4 &{} 0.1&{} 10.0 &{} 35.0 &{} 3.2&{} 0.0381 &{} 0.5743 &{}0.8828 \\ \end{matrix}$$			

Function	Dimensions	Range	$F_{min}$
	(N, T, dim)		
**F21 and F22 and F23:Shekel’s Familyl**			
$$F_{ij}\left( x\right) =-\sum _{i=1}^{5}{\sum _{j=1}^{dim}[(x_j-a_{ij})^2}+c_j]-1$$			
F21:Shekel’s 5 family	100,100,4	[0,10]	−5.0551
$$F_{21}\left( x\right) =-\sum _{i=1}^{5}{[(x-a_i)(x-a_i)^T}+c_i]-1$$			
Where, in this exercise: $$a=\begin{bmatrix} 4 &{} 4&{}4 &{} 4\\ 1 &{} 1&{}1 &{} 1\\ 8&{} 8&{}8 &{} 8\\ 6&{} 6&{}6 &{} 6\\ 3&{} 7&{}3 &{} 7\\ \end{bmatrix}$$ $$c=\begin{bmatrix}0.1\\ 0.2\\ 0.2\\ 0.4\\ 0.6\end{bmatrix}$$			
F22: Shekel’s 7 family	100,100,4	[0,10]	−5.088
$$F_{22}\left( x\right) =-\sum _{i=1}^{7}{[(x-a_i)(x-a_i)^T}+c_i]-1$$			
Where, in this exercise: $$a=\begin{bmatrix} 4&{}4 &{}4 &{}4 \\ 1&{} 1 &{}1 &{}1 \\ 8&{} 8&{} 8 &{}8 \\ 6&{} 6&{} 6 &{}6 \\ 3&{} 7&{} 3 &{}7 \\ 2&{} 9&{} 2 &{}9 \\ 5&{} 5&{} 3 &{}3 \\ \end{bmatrix}$$ $$c=\begin{bmatrix} 0.1\\ 0.2 \\ 0.2 \\ 0.4 \\ 0.4\\ 0.6 \\ 0.3\\ \end{bmatrix}$$			
F23:Shekel’s 10 family	100,100,4	[0,10]	−5.128
$$F_{23}\left( x\right) =-\sum _{i=1}^{10}{[(x-a_i)(x-a_i)^T}+c_i]-1$$			
Where, in this exercise: $$a=\begin{bmatrix} 4&{} 4 &{}4 &{} 4\\ 1&{}1 &{} 1 &{} 1\\ 8 &{} 8 &{} 8 &{}8 \\ 6 &{} 6 &{} 6 &{}6\\ 3 &{} 7 &{} 3&{} 7\\ 2 &{} 9 &{} 2 &{} 9\\ 5 &{}5&{} 3 &{}3\\ 8&{} 1 &{} 8 &{}1 \\ 6 &{} 2 &{} 6 &{}2 \\ 7 &{}3.6 &{}7 &{}3.6 \\ \end{bmatrix}$$ $$c=\begin{bmatrix} 0.1\\ 0.2 \\ 0.2 \\ 0.4\\ 0.4\\ 0.6 \\ 0.3\\ 0.7\\ 0.5\\ 0.5\\ \end{bmatrix}$$			

### Comparing LFGOA with AHA, AO, DA, DMOA, GBO, HGS, HHO, and MVO

To comparing and evaluating the performance of the LFGOA on the well-known 23 mathematical benchmark functions, we select below the eight advanced well-known and the latest meta-heuristic algorithms respectively.

1. Artificial hummingbird algorithm (AHA)^29^,
2. Aquila Optimizer (AO)^30^,
3. Dragonfly algorithm (DA)^31^,
4. Dwarf Mongoose Optimization Algorithm (DMOA)^32^,
5. Gradient-based optimizer (GBO)^33^,
6. Hunger Games Search (HGS)^34^,
7. Harris hawks optimization (HHO)^35^,
8. Multi-Verse Optimizer (MVO)^36^.

In order to provide a fair comparison, the main controlling parameters of these algorithms all run 30 times on each of the benchmark function, number of search agents and maximum iteration are all equal to 100 respectively. In the experiments, the key parameters of these nine algorithms are set up as shown in Table 2.

**Table 2:** The setup of the parameters.

Algorithm	Parameters	Value
AHA	migration coefficient	2*n*
AO		$G_1=2\times rand-1$ $G_2=2\times (1-\frac{t}{T})$ $QF\left( t\right) =t^\frac{2\times r a n d-1}{{(1-T)}^2}$
	quality function used to equilibrium	
	the search strategies QF the slope from the	
	first location (1) to the last location (t)	
DA	inertia weight *w*	$w=max+t\times \left( \frac{max-min}{T}\right)$
		$$min=0.4,\ max=0.9$$
	separation weight *s*	$s=2\times rand\times [0.1-t(\frac{0.1}{T/2})]$
	alignment weight *a*	$a=2\times rand\times [0.1-t(\frac{0.1}{T/2})]$
	the cohesion weight *c*	$c=2\times rand\times [0.1-t(\frac{0.1}{T/2})]$
	food factor *f*	$f=2\times rand$
	enemy factor *e*	$e=0.1-t(\frac{0.1}{{T/2}})$
DMOA	convergence constant a	$a={(1-\frac{t}{T})}^{2\times \frac{t}{T}}$
GBO	$\beta$ the most significant parameter	$\beta =\beta _{min}+(\beta _{max}-\beta _{min})\times {(1-{(\frac{t}{T})}^3)}^2$
	in the GBO to balance the exploration	
	and exploitation searching processes	$\beta _{min}=0.2,\beta _{max}=1.2,\ pr=0.5$
HGS	convergence constant a	$a=2\times (1-\frac{t}{T})$
HHO	$E_0$ is the initial state of its energy, *E* indicates the escaping energy of the prey.	$E=2E_0\times (1-\frac{t}{T})$
LFGOA		$C=C_{max}-t\times \frac{C_{max}-C_{min}}{T}$
	convergence constant C	$C_{max}=1, C_{min}=0.00001$
MVO	travelling distance rate (TDR) $\in {[}0.6 1{]}$	$TDR=1-\frac{t^{1/p}}{T^{1/p}},\ p=6$

*t* Current iteration,* T* The maximal iteration.

In the following Tables, where best results are all marked in bold.

In addition, to check the differences and rankings between nine algorithms, another non parametric multiple comparison method is used to calculate the average ranking value by the Friedman test. When applying Friedman’s test, the best algorithm is the one that receives the lowest rank while the worst algorithm receives the highest rank. In order to assess the statistical performance of LFGOA and each other method on the 23 test suites, the average (or mean) and standard deviation values of the rank of each method were taken into account. The average and Std rankings of LFGOA in conjunction with other methods using Friedman’s test are summarized in Tables 3 and 4, respectively.

**Table 3:** The values of average and average Rank on nine algorithms.

F	Index	AHA	AO	DA	DMOA	GBO	HGS	HHO	LFGOA	MVO
F1	Average	2.65E+00	1.46E+02	1.36E+03	6.97E+02	7.83E+07	6.45E+02	9.34E+02	**3.06E-09**	7.17E+02
	Rank	2	3	8	5	9	4	7	**1**	6
F2	Average	3.93E−02	5.57E-01	9.15E+00	4.21E+00	3.43E+35	9.42E+07	1.88E+08	**9.21E-29**	6.37E+00
	Rank	2	3	6	4	9	7	8	**1**	5
F3	Average	2.38E+02	1.02E+02	3.76E+03	2.28E+03	9.40E+01	2.30E+03	1.51E+03	**7.95E-10**	9.03E+02
	Rank	4	3	9	7	2	8	6	**1**	5
F4	Average	1.41E−01	7.46E-01	2.77E+01	1.63E+01	4.17E+06	1.57E+00	1.61E+00	**6.59E-06**	8.98E+00
	Rank	2	3	8	7	9	4	5	**1**	6
F5	Average	7.12E+01	8.44E+04	1.61E+06	4.63E+05	2.57E+03	2.14E+06	2.02E+06	**4.00E+00**	8.94E+05
	Rank	2	4	7	5	3	9	8	**1**	6
F6	Average	1.13E+02	1.41E+02	2.45E+03	8.13E+02	6.81E+05	2.11E+03	8.03E+02	**1.25E+00**	5.66E+02
	Rank	2	3	8	6	9	7	5	**1**	4
F7	Average	6.74E−02	5.60E-02	7.09E-01	1.53E-01	1.03E+01	3.38E+00	1.48E+00	**5.55E-01**	2.46E-01
	Rank	2	3	8	6	9	7	5	**1**	4
F8	Average	-5.94E+03	-2.39E+03	-2.26E+03	-7.12E+12	5.11E+01	-1.20E+04	-1.20E+04	**-1.34E+00**	-2.20E+03
	Rank	6	5	4	9	2	7	8	**1**	3
F9	Average	4.13E+00	1.60E+00	5.73E+01	5.01E+01	1.56E+01	1.11E+01	9.63E+00	**0.00E+00**	4.75E+01
	Rank	3	2	9	8	6	5	4	**1**	7
F10	Average	2.90E−01	2.77E-01	1.09E+01	6.54E+00	3.24E-01	1.15E+00	6.42E-01	**9.81E-09**	6.25E+00
	Rank	3	2	9	8	4	6	5	**1**	7
F11	Average	2.86E−01	1.37E+00	2.48E+01	7.30E+00	4.95E-03	5.31E+00	5.76E+00	**1.51E-07**	7.25E+00
	Rank	3	4	9	8	2	5	6	**1**	7
F12	Average	**8.05E−01**	3.27E+05	4.29E+06	6.53E+05	2.31E+00	4.66E+06	4.26E+06	4.11E+00	1.63E+06
	Rank	**1**	4	8	5	2	9	7	3	6
F13	Average	4.41E+00	3.38E+05	3.73E+06	2.33E+06	6.65E+01	1.21E+07	1.00E+07	**5.02E-01**	3.40E+06
	Rank	2	4	7	5	3	9	8	**1**	6
F14	Average	1.97E+00	3.50E+00	1.22E+00	1.63E+00	**4.54E-01**	1.57E+00	2.16E+00	2.11E+01	1.62E+00
	Rank	6	8	2	5	**1**	3	7	9	4
F15	Average	2.92E−03	2.42E-03	6.09E-03	1.58E-03	**7.85E-04**	1.71E-03	4.71E-03	1.48E-01	2.91E-03
	Rank	6	4	8	2	**1**	3	7	9	5
F16	Average	-1.03E+00	-1.01E+00	-1.00E+00	-1.03E+00	-1.03E+00	-1.03E+00	-1.02E+00	**-9.94E-04**	-1.01E+00
	Rank	6	3	2	7	8	9	5	**1**	4
F17	Average	4.10E−01	5.22E-01	4.17E-01	4.13E-01	**4.04E-01**	4.16E-01	4.09E-01	5.55E+01	4.37E-01
	Rank	3	8	6	4	**1**	5	2	9	1
F18	Average	**3.11E+00**	3.55E+00	4.09E+00	3.16E+00	3.59E+00	3.13E+00	3.20E+00	6.00E+02	3.71E+00
	Rank	**1**	5	8	3	6	2	4	9	7
F19	Average	-3.86E+00	-3.75E+00	-3.85E+00	-3.86E+00	-3.86E+00	-3.86E+00	-3.83E+00	**-6.81E-02**	-3.84E+00
	Rank	6	2	5	7	8	9	3	**1**	4
F20	Average	-3.26E+00	-2.67E+00	-3.06E+00	-3.25E+00	-3.29E+00	-3.26E+00	-2.98E+00	**-5.15E-03**	-3.09E+00
	Rank	7	2	4	6	9	8	3	**1**	5
F21	Average	-7.79E+00	-9.99E+00	-6.84E+00	-9.09E+00	-4.98E+00	-7.51E+00	-4.87E+00	**-2.74E-01**	-6.15E+00
	Rank	7	9	5	8	3	6	2	**1**	4
F22	Average	-7.96E+00	-1.03E+01	-7.43E+00	-9.24E+00	-9.94E+00	-9.18E+00	-4.93E+00	**-2.94E-01**	-2.42E+00
	Rank	5	9	4	7	8	6	3	**1**	2
F23	Average	-8.32E+00	-1.04E+01	-7.14E+00	-8.72E+00	-4.85E+00	-9.98E+00	-4.94E+00	**-3.23E-01**	-6.08E+00
	Rank	6	9	5	7	2	8	3	**1**	4
Friedmans test		3.7826	4.4348	6.4783	6.0435	5.0435	6.3478	5.2609	2.4783	4.8696

The best of the comparison results are in [bold].

In Table 3, there are 17 out of 23 average values obtained by LFGOA algorithm, which are all less than those obtained by the other eight algorithms. From Table 3, it can be seen that the average searching quality of LFGOA is better than those of other methods.

From the statistical results of Table 3, it is clear that the LFGOA with the complete improvement strategies performs best with a Friedman test ranking value of 2.4783. All in all, there are 18 out of 23 average ranking first obtained by LFGOA, which are all more than those obtained by the other eight optimization algorithms. However, LFGOA gives unsatisfactory results in F14, F15, F17, and F18. The results show that LFGOA achieves the average ranking third in F12. The LFGOA performs the best among the nine algorithms, proving that the utilization of Levy Flight can effectively enhance the performance of the GOA algorithm.

**Table 4:** The standard deviations and Std rank on nine algorithms.

	Index	AHA			AO		DA		DMOA		GBO		HGS		HHO		LFGOA		MVO	
	Std.	1.69E+01			1.43E+03		2.43E+03		1.78E+03		4.95E+08		5.02E+03		7.37E+03		**1.25E-08**		1.89E+03	
F1	Rank	2			3		6		4		9		7		8		**1**		5	
	Std.	2.07E-01			5.17E+00		7.58E+00		8.06E+00		3.43E+36		9.42E+08		1.88E+09		**1.99E-28**		1.46E+01	
F2	Rank	2			3		4		5		9		7		8		**1**		6	
	Std.	2.13E+03			9.34E+02		4.18E+03		2.20E+03		4.74E+02		1.33E+04		9.35E+03		**4.05E-09**		2.27E+03	
F3	Rank	4			3		7		5		2		9		8		**1**		6	
	Std.	8.32E-01			6.23E+00		2.22E+01		1.18E+01		3.07E+07		1.08E+01		9.66E+00		**1.36E-05**		1.10E+01	
F4	Rank	2			3		8		7		9		5		4		**1**		6	
	Std.	4.20E+02			8.44E+05		6.66E+06		2.51E+06		1.36E+04		2.13E+07		1.77E+07		**1.64E-03**		3.96E+06	
F5	Rank	2			4		7		5		3		9		8		**1**		6	
	Std.	1.13E+02			1.41E+03		2.99E+03		2.23E+03		6.25E+06		1.05E+04		6.55E+03		**2.04E-02**		1.44E+03	
F6	Rank	2			3		6		5		9		8		7		**1**		4	
	Std.	5.96E-01			5.37E-01		1.79E+00		3.36E-01		2.56E+00		1.65E+01		1.35E+01		**2.87E-01**		9.85E-01	
F7	Rank	4			3		6		2		7		9		8		**1**		5	
	Std.	1.20E+03			4.22E+02		3.82E+02		2.53E+13		4.07E+02		1.17E+03		1.96E+03		**2.05E+01**		2.95E+02	
F8	Rank	7			5		3		9		4		6		8		**1**		2	
	Std.	2.44E+01			1.11E+01		3.44E+01		1.78E+01		1.12E+02		4.78E+01		4.94E+01		**0.00E+00**		1.58E+01	
F9	Rank	5			2		6		4		9		7		8		**1**		3	
	Std.	1.60E+00			1.93E+00		7.42E+00		5.05E+00		1.04E+00		3.53E+00		2.94E+00		**1.47E-08**		4.19E+00	
F10	Rank	3			4		9		8		2		6		5		**1**		7	
	Std.	2.63E+00			1.33E+01		2.56E+01		1.71E+01		4.95E-02		5.15E+01		4.76E+01		**1.20E-06**		1.82E+01	
F11	Rank	3			4		7		5		2		9		8		**1**		6	
	Std.	3.10E+00			3.27E+06		1.24E+07		4.82E+06		6.67E+00		4.66E+07		4.23E+07		**8.73E-02**		1.08E+07	
F12	Rank	2			4		7		5		3		9		8		**1**		6	
	Std.	1.48E+01			3.38E+06		1.44E+07		1.10E+07		3.70E+02		1.21E+08		9.32E+07		**9.20E-03**		2.26E+07	
F13	Rank	2			4		6		5		3		9		8		**1**		7	
	Std.	1.34E+00			1.59E+00		2.25E+00		1.46E+00		2.49E+00		1.92E+00		**1.05E+00**		5.90E+01		2.91E+00	
F14	Rank	2			4		6		3		7		5		**1**		9		8	
	Std.	7.43E-03			7.37E-03		2.15E-02		**1.77E-03**		1.87E-03		4.16E-03		3.19E-02		4.91E-03		8.15E-03	
F15	Rank	6			5		8		**1**		2		3		9		4		7	
	Std.	**1.98E-03**			2.72E-02		1.25E-01		7.10E-03		2.56E-02		2.63E-02		1.12E-01		7.49E-03		1.27E-01	
F16	Rank	**1**			6		8		2		4		5		7		3		9	
	Std.	**3.62E-02**			1.72E-01		9.71E-02		9.61E-02		3.65E-02		1.32E-01		7.35E-02		8.58E-01		1.44E-01	
F17	Rank	**1**			8		5		4		2		6		3		9		7	
	Std.	**4.88E-01**			5.43E-01		4.64E+00		1.08E+00		5.84E+00		9.35E-01		1.49E+00		6.66E+00		3.90E+00	
F18	Rank	**1**			2		7		4		8		3		5		9		6	
	Std.	6.50E-03			3.48E-02		2.18E-02		2.95E-02		3.10E-02		1.72E-02		7.80E-02		**1.50E-04**		6.82E-02	
F19	Rank	2			7		4		5		6		3		9		**1**		8	
	Std.	1.60E-01			1.32E-01		2.23E-01		2.09E-01		1.61E-01		2.20E-01		4.77E-02		**1.43E-04**		3.67E-01	
F20	Rank	4			3		8		6		5		7		2		**1**		9	
	Std.	1.73E+00			1.12E+00		3.62E+00		2.27E+00		3.96E-01		2.64E+00		5.42E-01		**1.44E-03**		2.71E+00	
F21	Rank	5			4		9		6		2		7		3		**1**		8	
	Std.	2.43E+00			1.02E+00		3.31E+00		2.28E+00		1.55E+00		2.71E+00		6.57E-01		**1.32E-02**		5.30E-01	
F22	Rank	7			4		9		6		5		8		3		**1**		2	
	Std.	1.64E+00			9.47E-01		3.28E+00		2.82E+00		8.53E-01		1.72E+00		6.13E-01		**2.45E-03**		3.39E+00	
F23	Rank	5			4		8		7		3		6		2		**1**		9	
Friedmans test		3.2174			4.0000		6.6957		4.9130		5.0000		6.6522		6.0870		2.2609		6.1739	

The best of the comparison results are in [bold].

In Table 4, only the composite functions F14–F18, the standard deviation value obtained by LFGOA algorithm are 5.90E+01, 4.91E−03, 7.49E−03, 8.58E−01, and 6.66E+00, which are not less than the other eight algorithms. All in all, there are 18 out of 23 standard deviation values obtained by LFGO algorithm, which are all less than those obtained by the other eight algorithms. The better values of the standard deviations prove that the LFGOA algorithm stable performs better than the other eight algorithms.

As shown in Table 4, we evaluate the performance of the algorithms using the Friedman test. All algorithms are ranked according to the Std value. LFGOA ranks first in all unimodal functions (F1–F7) and all multi-modal functions (F8–F13) and achieves a Std ranking value of 2.2609. However, LFGOA gives unsatisfactory results in F14, F17, and F18. In this regard, the results show that LFGOA achieves the Std ranking third in F16 and the fourth in F15. The statistical results show that LFGOA has the best performance compared to the eight algorithms mentioned above for solving the 23 classical test functions.

**Table 5:** The best values of AHA, AO, DA, DMOA, GBO, HGS, HHO, LFGOA, and MVO.

F	AHA	AO	DA	DMOA	GBO	HGS	HHO	LFGOA	MVO
F1	5.27E-34	1.99E-39	4.47E-02	3.84E-02	7.49E-17	**0.00E+00**	6.22E-33	2.20E-11	1.20E-01
F2	4.34E-15	7.20E-20	5.21E-01	2.10E-02	**1.20E-33**	1.01E-26	2.72E-20	1.94E-31	8.16E-02
F3	3.32E-29	**1.95E-39**	1.20E+01	3.49E+02	1.61E-22	1.45E-38	4.74E-27	1.19E-12	2.91E-01
F4	2.48E-14	2.72E-20	3.99E+00	4.13E+00	2.63E+01	**3.20E-28**	2.25E-15	5.18E-07	1.64E-01
F5	2.87E+01	1.28E-04	1.01E+01	1.47E+02	1.05E-22	**2.28E-02**	4.26E-02	3.84E+00	5.99E+01
F6	**0.00E+00**	2.20E-06	1.52E+00	1.94E-02	2.02E-20	6.45E-06	3.88E-04	2.31E-01	1.19E-01
F7	3.63E-04	1.91E-04	7.03E-03	8.41E-03	2.18E+00	**1.10E-04**	7.29E-04	1.31E-04	3.82E-03
F8	−7.51E+03	−3.01E+03	−2.51E+03	−1.38E+14	2.25E-02	**−1.26E+04**	−1.26E+04	−1.58E+03	−2.57E+03
F9	0.00E+00	0.00E+00	1.64E+01	3.42E+01	3.82E-04	**0.00E+00**	0.00E+00	0.00E+00	3.79E+01
F10	4.44E-15	8.88E-16	2.35E+00	5.68E-01	7.90E-14	**8.88E-16**	4.44E-15	1.45E-09	1.86E-01
F11	0.00E+00	0.00E+00	7.60E-01	7.01E-01	2.42E-13	**0.00E+00**	0.00E+00	3.51E-10	7.12E-01
F12	6.31E-02	**4.23E-08**	3.75E-01	2.46E-01	5.73E-01	3.19E-06	8.01E-06	9.23E-05	3.79E-03
F13	2.71E+00	**7.88E-07**	1.55E-02	1.51E-01	4.81E-01	1.92E-06	3.46E-05	1.97E-05	3.54E-02
F14	**9.98E-01**	2.98E+00	**9.98E-01**	**9.98E-01**	1.01E-14	**9.98E-01**	1.99E+00	**9.98E-01**	**9.98E-01**
F15	4.17E-04	4.87E-04	1.66E-03	1.06E-03	**3.07E-04**	7.78E-04	3.25E-04	1.31E-03	7.55E-04
F16	−1.03E+00	−1.03E+00	−1.03E+00	−1.03E+00	−1.03E+00	−1.03E+00	−1.03E+00	−1.03E+00	−1.03E+00
F17	3.98E-01	3.98E-01	3.98E-01	3.98E-01	3.98E-01	3.98E-01	3.98E-01	3.98E-01	3.98E-01
F18	3.00E+00	3.02E+00	3.00E+00	3.00E+00	3.00E+00	3.00E+00	3.00E+00	3.00E+00	3.00E+00
F19	**−3.86E+00**	**−3.85E+00**	**−3.86E+00**	**−3.86E+00**	**−3.86E+00**	**−3.86E+00**	**−3.86E+00**	−3.59E+00	**−3.86E+00**
F20	**−3.32E+00**	−3.08E+00	−3.17E+00	**−3.32E+00**	**−3.32E+00**	**−3.32E+00**	−3.02E+00	**−3.32E+00**	**−3.32E+00**
F21	−9.61E+00	−1.02E+01	−1.02E+01	−1.02E+01	−5.06E+00	−1.02E+01	−5.05E+00	**−5.06E+00**	−1.02E+01
F22	−1.04E+01	−1.04E+01	−1.04E+01	−1.04E+01	−1.04E+01	−1.04E+01	**−5.09E+00**	**−5.09E+00**	−2.77E+00
F23	−1.04E+01	−1.05E+01	−1.05E+01	−1.05E+01	−5.13E+00	−1.05E+01	−5.11E+00	**−5.13E+00**	−1.05E+01

The best of the comparison results are in [bold].

In Table 5, for the unimodal functions and the multimodal functions, the best values obtained by the LFGOA algorithm are not desired in comparison with other eight algorithms. For the composite functions, only the F15, the LFGOA algorithms get nearly accurate approximation values, for the other composite functions F14 and F16–F23, the LFGOA algorithm all get better accurate approximation values.

To further analyze the differences between the algorithms, a post-hoc Nemenyi test was employed. If the null-hypothesis is rejected, we can proceed with a post-hoc test. The Nemenyi test (Nemenyi, 1963) is similar to the Tukey test for ANOVA and is used when all classifiers are compared to each other. The performance of two classifiers is significantly different if the corresponding average ranks differ by at least the critical difference (CD).

15 $$\begin{aligned} CD=q_\alpha \sqrt{\frac{k(k+1)}{6N}} \end{aligned}$$

where *N* is the number of datasets (23) and *k* (9) is the number of algorithms being compared.

**Table 6:** Critical values for post-hoc tests after the Friedman test.

#classifiers	2	3	4	5	6	7	8	9	10
q0.05	1.96	2.343	2.569	2.728	2.85	2.949	3.031	3.102	3.164
q0.10	1.645	2.052	2.291	2.459	2.589	2.693	2.78	2.855	2.92

At $\alpha =0.05$, the critical value (Table 6) $q_\alpha$ for 9 classifiers (algorithms) is 3.102 and the corresponding CD is $3.102 \times \sqrt{\frac{9 \times 10}{6 \times 23}} \approx 2.5051$.

At $\alpha =0.10$, $q_\alpha =2.855$, $N=23$, $k=9$; corresponding CD is $2.855 \times \sqrt{\frac{9 \times 10}{6 \times 23}} \approx 2.3056$.

To find differences in nine algorithms, critical difference (CD) based on the Nemenyi test was used. The critical value $q_\alpha$ is 3.102, so the CD is 2.5051. A post-hoc test concludes that if the difference in Friedman ranking values between the two algorithms is less than the CD value, there is no significant difference between the two algorithms; conversely, there is a significant difference between the two algorithms.

In Table 7, the “Diff with LFGOA” in the third column indicates the differences in average rank between LFGOA and other eight algorithms, and the “Diff with LFGOA” in the fifth column indicates the differences in Std rank between LFGOA and other eight algorithms respectively.

**Figure 3:**
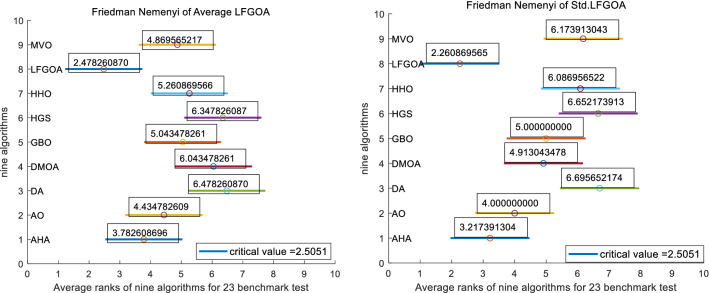
Average rank of Mean (left side) and Average rank of Std (right side) about LFGOA and the other algorithms using Friedman’s test based upon their results on the 23 test functions.

Critical Difference (CD) diagrams in Fig. 3 are simple and intuitive visualizations of the results of a Nemenyi post-hoc test that is designed to check the statistical significance between the differences in average rank of a set of nine algorithms respectively on a set of 23 benchmark test functions.

Fig. 3 shows the analysis results of the data from Table 7. In each line segments, we plot the average ranks about mean (left side in the Fig. 3) and Std (right side in the Fig. 3) of nine algorithms. The length of the line segment indicates the CD value, and the center of each line segment labeled “circle mark” represents the value of the average rank position about mean (left side) and Std (right side) of the respective each algorithm across all 23 benchmark test functions. If the value of the center between two line-segments (intervals) is greater than the CD, it means that the two algorithms do not overlap each other, which indicate there is a statistically significant difference between them.

As shown in Fig. 3, LFGOA ranks first (The best average ranks are to the left side) in Mean and Std respectively. From the Fig. 3 and Table 7, we can clear see that LFGOA versus AO and LFGOA versus AHA have similar performance in terms of average ranks of Mean and Std.

**Table 7:** The Average and Std ranks of nine algorithms using Friedman’s test based upon their results on the 23 test functions.

Algorithm	Average		Std	
	Mean rank	Diff with LFGOA	Std Rank	Diff with LFGOA
AHA	3.782608696	1.3043 < 2.5051	3.217391304	0.9565 < 2.5051
AO	4.434782609	1.9565 < 2.5051	4.000000000	1.7391 < 2.5051
DA	6.478260870	4.0000 > 2.5051	6.695652174	4.4348 > 2.5051
DMOA	6.043478261	3.5652 > 2.5051	4.913043478	2.6522 > 2.5051
GBO	5.043478261	2.5652 > 2.5051	5.000000000	2.7391 > 2.5051
HGS	6.347826087	3.8696 > 2.5051	6.652173913	4.3913 > 2.5051
HHO	5.260869566	2.7826 > 2.5051	6.086956522	3.8261 > 2.5051
LFGOA	2.478260870	0	2.260869565	0
MVO	4.869565217	2.3913 < 2.5051	6.173913043	3.9130 > 2.5051

### Comparing the performance of LFGOA with AHA, AO, DA, DMOA, GBO, HGS, HHO, and MVO algorithm

**Figure 4:**
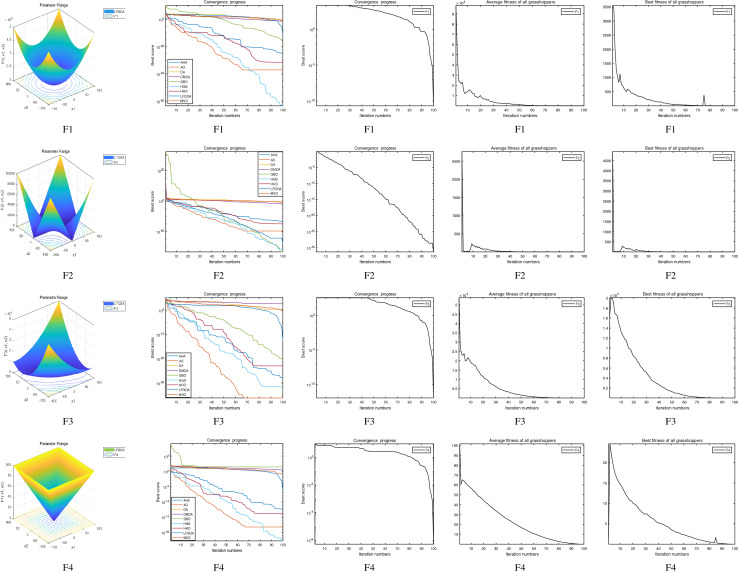

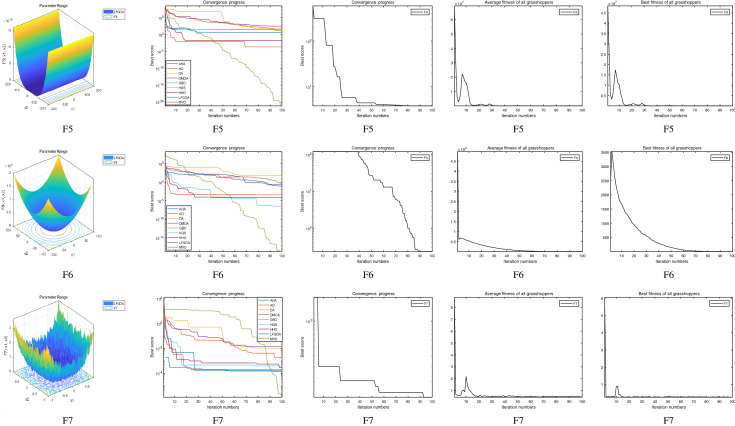
The performance of LFGOA in the unimodal test functions F1–F7.

**Figure 5:**
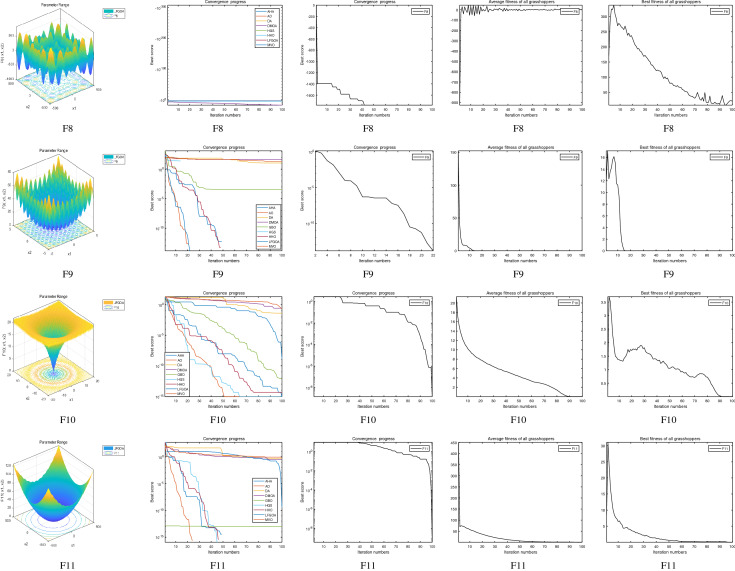
The performance of LFGOA in the multimodal test functions F8–F13.

**Figure 6:**
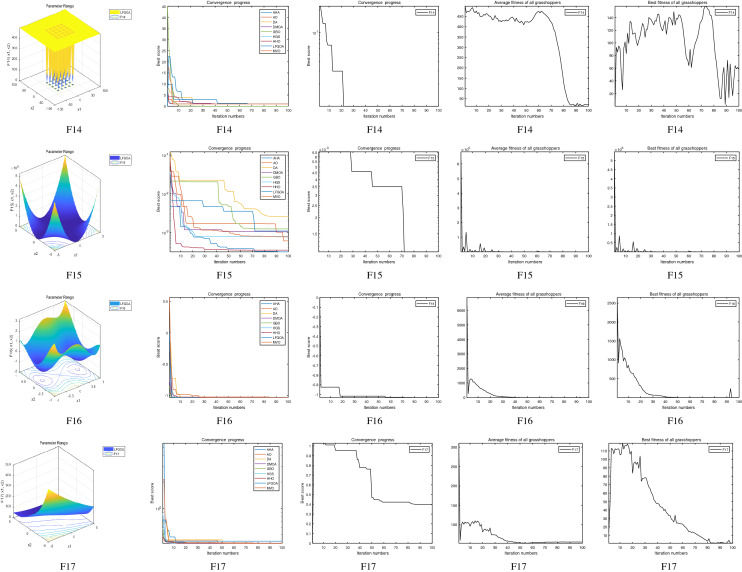

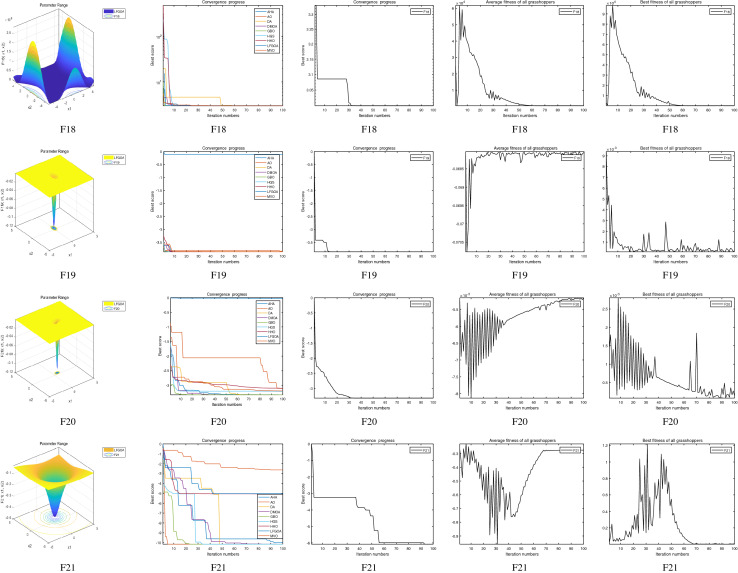

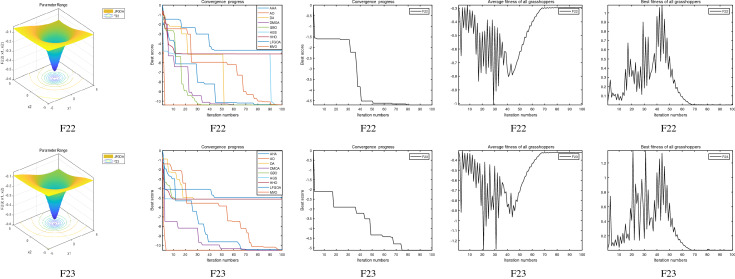
The performance of LFGOA in the composite test functions F14–F23.

The performance of the AHA, AO, DA, DMOA, GBO, HGS, HHO, LFGOA, and MVO algorithms are respectively benchmarked in the following figures. In the first column in Fig. 4, Fig. 5, and Fig. 6, the graph is a three-dimensional drawing of the cost function. The second column of the Fig. 4, Fig. 5, and Fig. 6, the graph shows the independently convergence progress of the AHA, AO, DA, DMOA, GBO, HGS, HHO, LFGOA, and MVO algorithms respectively. The third column of the Fig. 4, Fig. 5, and Fig. 6, the graph focus on the convergence progress of the LFGOA algorithm on each of the F1–F23 benchmark functions respectively. The fourth column of the Fig. 4, Fig. 5, and Fig. 6, the graph focus on the average fitness history of the LFGOA algorithm on each of the F1–F23 benchmark functions respectively. The fifth column of the Fig. 4, Fig. 5, and Fig. 6, the graph focus on the best fitness history of the LFGOA algorithm on each of the F1–F23 benchmark functions respectively.

#### The unimodal test functions F1–F7

Since there is only one extreme point in F1–F7 unimodal benchmark functions, the unimodal benchmark functions are suitable for assessing the convergence rate and benchmarking the exploitation behavior of the algorithm. In the second column in Fig. 4, the LFGOA algorithm shows the best results in 6 out of 7, especially on F1–F4 respectively in unimodal benchmark, but for F5, the result unsatisfactory for the LFGOA algorithm. In unimodal functions of F6–F7, the GBO algorithm shows better result that nearly reaches to zero.

#### The multimodal test functions F8–F13

The F8–F13 multimodal benchmark functions are used to assess the exploration capability of the LFGOA algorithm to find global optima when the number of local optima increases exponentially with the problem dimension.

The second column of Fig. 5, for F8, as the GBO algorithm present a wrong value of positive (reference the Table 5) against the value of negative that gotten by the AHA, AO, DA, DMOA, HGS, HHO, LFGOA, and MVO algorithms respectively, the figure only shows the best convergence progress of the AHA, AO, DA, DMOA, HGS, HHO, LFGOA, and MVO algorithms respectively without GBO, because great difference values on two directions can’t be appropriately plotted in the same figure. For F9–F13, the convergence progress of the LFGOA algorithm is satisfactory especially for F9–F11; the convergence rate of the LFGOA algorithm is rapidly. Since the multimodal functions have an exponential number of local solutions, the results show that the LFGOA algorithm can explore the search space extensively and find promising regions of the search space.

For the third column of Fig. 5, the convergence progress of the LFGOA algorithm on each of the F8–F13 benchmark functions all exhibit excellent convergence rate on each of the F8–F13 benchmark functions. It can also be seen in the third column of the Fig. 5, that the LFGOA algorithm does not provide uniform convergence behavior in all the benchmark functions. This shows that the LFGOA algorithm is good in handling of different problems.

#### The composite test functions F14–F23

The second column of Fig. 6, for F14–F20, all of the algorithms reached the satisfactory convergence rate. For F21 and F23 (reference the Table 5), only the final results of the convergence progress of the GBO and LFGOA algorithms respectively are satisfactory, the other seven algorithms unsatisfactory. For F22 (reference the Table 5), only the final results of the convergence progress of the HHO and LFGOA algorithms respectively are satisfactory, the other seven algorithms unsatisfactory. All in all, for the composite benchmark functions of F14–F23, the comprehensive result of the convergence progress of the LFGOA algorithm is superior to the other algorithms, which is very similar to the situation of the Table 5. From the third column of the Fig. 6, the convergence progress of the LFGOA on each of the F14–F23 benchmark functions all exhibit better convergence rate. For the fourth column of the Fig. 6, even the average fitness of all grasshoppers on the F20–F23 with high fluctuation during the exploration phase (at nearly the early iteration stage) and low changes in the exploitation phase (at the end of iteration stage). This proves that the LFGOA algorithm is able to eventually improve the fitness of initial random solutions for a given optimization problem. For the fifth column of the Fig. 6, even the best fitness of all grasshoppers on the F14 and F20–F23 with high fluctuation during the exploration phase (at nearly the early iteration stage) and low changes in the exploitation phase (at the end of iteration stage), which guarantee that the LFGOA algorithm exploration extensively over the initial stage and exploitation locally at the end of optimization, and eventually convergences to optimization points.

### LFGOA vs the other eight optimization algorithms on the *p-*Values of the wilcoxon

Due to the stochastic nature of the algorithms, the averages and standard deviation only compare the overall performance of the algorithms, while a statistical test considers each run’s results and proves that the results are statistically significant. Derrac et al^37^, recommended that to evaluate the performance of algorithms, statistical tests should be done. The non-parametric Wilcoxon statistical test is conducted and the *p-*values that are less than 0.05 could be considered as strong evidence against the null hypothesis. To assess the overall performance of the LFGOA algorithm, and to confirm the significance and robustness of the results, we apply Wilcoxon’s statistical test with a 5% significance level to the obtained average accuracy results.

**Table 8:** The *p-*Values of the Wilcoxon rank-sum test over 23 benchmark functions.

F	AHA	AO	DA	DMOA	GBO	HGS	HHO	MVO
F1	1.33E-29	1.12E-30	1.10E-03	1.36E-04	4.92E-04	1.66E-27	3.81E-29	1.07E-02
F2	9.55E-05	2.89E-01	1.72E-34	2.39E-31	3.18E-01	1.65E-01	5.83E-04	5.94E-34
F3	3.71E-26	2.20E-29	9.50E-09	1.83E-34	1.52E-14	1.24E-23	4.49E-19	3.11E-07
F4	2.00E-30	5.28E-31	9.68E-01	4.04E-11	2.07E-34	8.90E-30	8.74E-27	1.83E-01
F5	5.63E-15	1.06E-28	6.79E-29	2.17E-34	4.57E-09	8.19E-15	6.66E-21	5.05E-31
F6	3.07E-34	1.76E-32	3.17E-12	6.34E-01	3.17E-08	1.93E-26	1.01E-28	8.20E-03
F7	1.14E-02	1.30E-03	9.41E-35	8.51E-35	4.41E-23	3.21E-31	7.59E-30	2.60E-34
F8	7.48E-39	1.34E-36	7.30E-39	8.07E-39	8.08E-39	7.95E-39	8.06E-39	4.71E-39
F9	2.15E-05	9.75E-01	1.01E-36	2.79E-38	4.14E-31	4.55E-01	2.44E-05	1.15E-36
F10	2.62E-23	9.46E-27	6.68E-09	9.96E-18	3.73E-11	4.66E-23	7.46E-24	1.71E-28
F11	1.53E-29	7.68E-33	9.95E-04	1.68E-01	2.57E-34	7.94E-28	3.83E-27	2.63E-01
F12	1.85E-04	7.78E-35	3.61E-13	3.93E-16	3.70E-03	5.03E-32	5.03E-32	5.71E-16
F13	1.76E-34	2.18E-33	2.72E-13	2.11E-23	4.80E-09	3.18E-24	1.97E-27	9.16E-15
F14	1.56E-16	2.73E-01	1.34E-01	1.29E-05	5.18E-26	4.46E-32	8.75E-29	2.08E-28
F15	1.27E-24	2.31E-04	9.33E-10	7.59E-14	9.82E-04	2.00E-19	7.58E-29	7.45E-12
F16	3.80E-19	7.14E-33	4.09E-27	3.11E-29	2.82E-33	2.05E-30	5.70E-28	8.30E-08
F17	5.99E-12	6.12E-08	9.03E-01	1.05E-24	1.52E-20	9.27E-26	2.03E-21	7.26E-01
F18	3.38E-11	3.17E-41	3.94E-02	1.19E-25	7.84E-36	4.45E-26	1.09E-26	2.20E-03
F19	7.38E-39	1.21E-40	4.98E-39	7.74E-39	8.06E-39	8.09E-39	8.10E-39	7.54E-39
F20	1.77E-35	5.95E-37	1.26E-35	1.82E-35	1.88E-35	1.89E-35	1.89E-35	1.78E-35
F21	3.67E-22	3.59E-36	4.94E-06	3.29E-17	2.56E-31	1.25E-25	3.58E-20	6.52E-23
F22	6.72E-26	3.38E-38	6.45E-16	2.06E-29	9.21E-25	6.63E-34	6.22E-26	7.86E-16
F23	1.59E-22	2.73E-38	1.48E-12	8.93E-33	9.58E-25	6.51E-33	2.71E-28	4.35E-14

At Table 8, the *P-*values are more than 0.05 appeared in the following cases:

- LFGOA/AO in F2, F9, and F14 in the third column of Table 8.
- LFGOA/DA, the F4, F14, and F17 in the fourth column of Table 8, as above depicted, both the DA and LFGOA algorithms all embedded Levy Flight mechanism, which means both of the two algorithms have some extent similarity properties.
- LFGOA/DMOA in F6 and F11 in the fiveth column of Table 8.
- LFGOA/GBO in the F2 in the sixth column of Table 8.
- LFGOA/HGS, which is consistent with the F2 and H9 in the seventh column of Table 8.
- LFGOA/MVO, in the F4, F11, and F17, as above depicted, both the exploration and exploitation swarming behaviors of MVO are very similar to LFGOA.

The results of the *p-*values in Table 8 show that the superiority of the LFGOA algorithm is statistically significant.

### The scalability test for LFGOA

Comparing comprehensive and thoroughly the property of the LFGOA algorithm with the GOA algorithm, we conducted the scalability test here. As we known, the scalability test can help us to some extent understand the impact of the dimension on the capability of the solution and the effectively of the LFGOA algorithm. An in-depth exploration of the impacts on the solution functionality to catch what appears for the features of the LFGOA and GOA algorithms as the dimension of function experiences a growth respectively. Therefore, four dimensions of the functions F1–F23 are used here: 50, 100, 300, and 500. The whole circumstances have remained consistent; each algorithm uses 100 search agents and runs 30 times respectively. The mean values, the standard deviation values and the best optimal values were picked by the LFGOA and GOA algorithms under 50, 100, 300, and 500 dimensions, which are shown in the following tables.

**Table 9:** The average values and consumed time under dimensions are equal to 50 and 100.

	Average (D=50)					Average (D=100)					
	GOA	Time	LFGOA	Time		GOA	Time		LFGOA	Time	
F1	3.6191E+01	**1.2050**	**2.4942E-08**		1.2275	1.2055E+00		2.3869	**3.0569E-09**		2.9643
F2	1.3482E-03	**1.0951**	**5.7176E-20**		1.1334	2.4131E-05		2.2161	**9.2086E-29**		3.0393
F3	2.3206E-03	1.1223	**6.7550E-09**		**1.1118**	5.8872E-03		2.2003	**7.9485E-10**		2.4450
F4	6.5617E-02	**1.1066**	**5.7340E-05**		1.1322	1.5523E-02		2.2044	**6.5922E-06**		2.5821
F5	**1.7916E+00**	**1.1356**	3.9995E+00		1.1487	3.8084E+00		2.2154	3.9998E+00		2.6185
F6	**3.9447E-03**	1.1237	1.2511E+00		**1.0964**	3.5299E-04		2.1987	1.2513E+00		2.5916
F7	5.4859E-01	**1.1118**	**5.1284E-01**		1.1538	5.0714E-01		2.2203	5.5455E-01		2.5345
F8	-1.5817E+03	**1.1047**	**8.8354E-01**		1.1323	-1.7396E+03		2.2013	**-1.3428E+00**		2.5719
F9	9.9496E+00	**1.0977**	**0.0000E+00**		1.1305	2.0894E+01		2.1709	**0.0000E+00**		2.6047
F10	2.9110E-02	1.1078	**1.6046E-08**		**1.1006**	1.8809E-04		2.2013	**9.8080E-09**		2.5264
F11	2.8866E-01	1.1249	**6.8659E-06**		**1.1194**	2.1058E-01		2.2216	**1.5094E-07**		2.6239
F12	**9.0110E-04**	**1.1309**	4.1278E+00		1.1495	7.2328E-05		2.2531	4.1089E+00		2.6204
F13	**2.8781E-04**	**1.1260**	5.0433E-01		1.1367	2.0603E-04		2.2669	5.0215E-01		2.6213
F14	**9.9800E-01**	1.2246	1.6838E+01		**1.1961**	9.9800E-01		2.3730	2.1102E+01		2.7878
F15	**1.6420E-03**	**1.0955**	1.4814E-01		1.1130	1.6133E-03		2.1531	1.4768E-01		2.5356
F16	−1.0316E+00	1.1029	**−2.1599E-04**		**1.0986**	−1.0316E+00		2.1633	**−9.9360E-04**		2.5900
F17	**3.9789E-01**	**1.0719**	5.5426E+01		1.0845	3.9789E-01		2.1448	5.5550E+01		2.5279
F18	**3.0000E+00**	**1.0768**	6.0187E+02		1.0914	3.0000E+00		2.1536	6.0022E+02		2.5008
F19	-3.8556E+00	**1.1103**	**−6.8414E-02**		1.1153	−3.8628E+00		2.1828	**−6.8054E-02**		2.5666
F20	−3.3220E+00	**1.1251**	**−5.1192E-03**		1.1571	−3.2031E+00		2.1761	**−5.1526E-03**		2.5948
F21	−1.0153E+01	1.1073	**−2.7445E-01**		**1.1072**	−1.0153E+01		2.2019	**−2.7443E-01**		2.5493
F22	−1.0403E+01	**1.1054**	**−2.9499E-01**		1.1164	−1.0403E+01		2.1760	**−2.9359E-01**		2.5634
F23	−5.1756E+00	1.1077	**−3.2385E-01**		**1.0913**	−1.0536E+01		2.1356	**−3.2322E-01**		2.5238

The best of the comparison results are in [bold].

In Table 9 (D = 50), there are 15 out of 23 average values obtained by the LFGOA algorithm, which are all less than those obtained by the GOA algorithm.

In Table 9 (D = 100), there are 14 out of 23 average values obtained by the LFGOA algorithm, which are all less than those obtained by the GOA algorithm. Table 9 also tell us the LFGOA algorithm consumed a little more time than the GOA algorithm under dimensions equal to 50 and 100 respectively.

**Table 10:** The average values and consumed time under dimensions are equal to 300 and 500.

	Average (D=300)				Average (D=500)			
	GOA	Time	LFGOA	Time	GOA	Time	LFGOA	Time
F1	1.3993E-01	**7.0597**	**3.2786E-11**	7.1007	4.4886E-02	**11.6255**	**6.3759E-12**	11.6318
F2	1.0234E-04	**6.4803**	**5.0309E-65**	6.6171	3.8426E-05	10.7531	**4.5746E-83**	**10.7092**
F3	3.4992E-07	6.5596	**2.1009E-11**	**6.5256**	2.8104E-09	**10.8761**	**1.6953E-12**	10.8769
F4	6.1214E-05	**6.5041**	**7.4812E-07**	6.6153	2.2518E-05	**10.6880**	**2.2156E-07**	10.8303
F5	2.2175E+01	6.4526	**4.0006E+00**	**6.6215**	**4.4415E-01**	**10.7493**	4.0267E+00	10.8675
F6	**9.6501E-08**	**6.4276**	1.2483E+00	6.6123	**6.1274E-09**	**10.7097**	1.2499E+00	10.7890
F7	**4.7933E-01**	**6.4718**	4.7956E-01	6.7439	**5.3289E-01**	**10.7331**	4.8982E-01	10.7668
F8	−1.5422E+03	**6.5236**	**1.4705E-01**	6.6725	−1.8565E+03	10.8361	**−6.3545E-01**	**10.8322**
F9	1.1940E+01	**6.5637**	**0.0000E+00**	6.5827	3.9798E+00	**10.6932**	**0.0000E+00**	10.8322
F10	2.8382E-05	**6.4535**	**1.4523E-08**	6.6826	1.3150E-05	**10.7786**	**2.0263E-09**	10.8077
F11	1.0846E-01	**6.5031**	**2.7470E-11**	6.7343	2.9332E-01	**10.8807**	**9.4160E-12**	10.9145
F12	**6.9024E-09**	**6.5605**	4.1202E+00	6.7251	**1.3700E-10**	11.1410	4.1246E+00	**11.0573**
F13	**5.2347E-09**	**6.5703**	5.0618E-01	6.8489	**2.7928E-09**	11.1144	5.0520E-01	**11.0723**
F14	**9.9800E-01**	7.1762	2.7061E+01	**7.1094**	**9.9800E-01**	**11.8381**	1.4163E+01	11.9388
F15	**2.0363E-02**	**6.3858**	1.4785E-01	6.5066	**1.2232E-03**	**10.5673**	1.4900E-01	10.6402
F16	-1.0316E+00	**6.3136**	**-1.5066E-04**	6.5308	-1.0316E+00	**10.4830**	**-5.5530E-04**	10.6101
F17	**3.9789E-01**	**6.3308**	5.5468E+01	6.4903	**3.9795E-01**	**10.5142**	5.5418E+01	10.6483
F18	**3.0000E+00**	**6.3583**	6.0081E+02	6.4867	**3.0000E+00**	**10.4125**	2.8383E+03	10.7202
F19	−3.8628E+00	**6.3754**	**−6.8537E-02**	6.6820	−3.8628E+00	10.8593	**−6.8651E-02**	**10.7968**
F20	−3.3220E+00	**6.3398**	**−5.1478E-03**	6.5773	−3.3220E+00	**10.5832**	**−5.1397E-03**	10.7650
F21	−1.0153E+01	**6.4471**	**−2.7419E-01**	6.5988	−1.0153E+01	**10.6039**	**−2.7433E-01**	10.6293
F22	−1.0403E+01	**6.3645**	**−2.9496E-01**	6.6207	−5.0877E+00	**10.5663**	**−2.9534E-01**	10.6410
F23	−5.1756E+00	**6.4597**	**−3.2337E-01**	6.6613	−5.0877E+00	**10.5663**	**−3.2345E-01**	10.9356

The best of the comparison results are in [bold].

In Table 10 (D = 300), there are 15 out of 23 average values obtained by the LFGOA algorithm, which are all less than those obtained by the GOA algorithm.

In Table 10 (D = 500), there are 14 out of 23 average values obtained by the LFGOA algorithm, which are all less than those obtained by the GOA algorithm. Table 10 also tell us the LFGOA algorithm consumed a little more time than GOA under dimensions equal to 300 and 500 respectively.

**Table 11:** The Std values of dimensions are equal to 50 and 100.

	Std.(D=50)		Std.(D=100)	
	GOA	LFGOA	GOA	LFGOA
F1	6.0155E-08	**3.8798E-08**	1.4776E-08	**1.2465E-08**
F2	2.3807E-09	**1.1745E-19**	7.1900E-19	**1.9946E-28**
F3	**9.3962E-10**	2.6617E-08	1.5437E-09	4.0466E-09
F4	**1.1334E-08**	1.0041E-04	1.5319E-08	1.3578E-05
F5	**1.1839E-09**	5.4491E-03	1.9185E-09	1.6444E-03
F6	**1.4169E-09**	2.2225E-02	5.7538E-10	2.0425E-02
F7	**2.5696E-01**	2.7239E-01	3.0056E-01	**2.8663E-01**
F8	**6.7393E-09**	9.0843E+00	**1.2751E-09**	2.0548E+01
F9	**4.0619E-11**	0.0000E+00	**2.1905E-13**	0.0000E+00
F10	**2.7113E-09**	1.6339E-08	**2.2388E-09**	1.4696E-08
F11	**4.4558E-09**	5.0581E-05	**6.4376E-10**	1.2040E-06
F12	4.6808E-11	**1.3597E-11**	**2.1111E-11**	**1.6469E-11**
F13	**1.9813E-11**	3.0507E-10	**3.1630E-11**	**1.3907E-11**
F14	**8.6804E-16**	1.6792E-15	**8.6517E-16**	1.6865E-15
F15	**3.2066E-13**	1.8154E-12	**3.8729E-13**	5.5304E-13
F16	**3.0710E-13**	1.0826E-12	**3.2260E-13**	**1.7963E-13**
F17	6.1888E-12	**1.4536E-12**	**9.7940E-13**	**2.7986E-13**
F18	1.1610E-11	**6.8103E-12**	**1.6063E-12**	1.1737E-11
F19	8.9265E-15	**1.7853E-15**	**3.5706E-15**	8.0339E-15
F20	**4.4562E-13**	1.1664E-10	**8.0186E-14**	**5.9709E-14**
F21	3.4335E-11	**1.8687E-11**	2.3315E-11	**2.0967E-12**
F22	5.2950E-11	**2.5783E-11**	**1.0470E-11**	**2.7518E-12**
F23	**1.6863E-11**	2.4249E-11	**1.2424E-11**	**2.6440E-12**

The best of the comparison results are in [bold].

In Table 11 (D = 50), only for the unimodal functions F1 and F2, the Std values obtained by the LFGOA algorithm are 3.8798E-08 and 1.1745E-19, for the multimodal function F12, the Std values obtained by the LFGOA algorithm is 1.3597E-11, for the composite functions F17–F19 and F21–F22, the Std values obtained by the LFGOA algorithm are 1.4536E-12, 6.8103E-12, 1.7853E-15, 1.8687E-11, and 2.5783E-11, which are less than the GOA algorithm.

In Table 11 (D = 100), for the unimodal functions F1, F2 and F7, the Std values obtained by the LFGOA algorithm are 1.2465E-08, 1.9946E-28, and 2.8663E-01, for the multimodal functions F12 and F13, the Std values obtained by the LFGOA algorithm are 1.6469E-11 and 1.3907E-11, for the composite functions F16–F17 and F20–F23, the Std values obtained by the LFGOA algorithm are 1.7963E-13, 2.7986E-13, 5.9709E-14, 2.0967E-12, 2.7518E-12, and 2.6440E-12, which are less than the GOA algorithm.

**Table 12:** The Std values of dimensions are equal to 300 and 500.

	Std.(D=300)		Std.(D=500)	
	GOA	LFGOA	GOA	LFGOA
F1	5.4447E-09	**1.3549E-10**	3.0741E-09	**3.0527E-11**
F2	1.1990E-12	**1.1144E-64**	5.4483E-20	**1.4144E-82**
F3	**1.5002E-11**	8.2788E-11	**2.3827E-12**	4.3344E-12
F4	**1.7313E-08**	1.0348E-06	**1.5959E-08**	2.7880E-07
F5	**8.5595E-11**	5.7502E-03	**1.2438E-09**	2.0807E-01
F6	**1.0759E-11**	2.4281E-02	**2.7215E-12**	1.4023E-02
F7	**2.7826E-01**	2.9240E-01	2.9060E-01	**2.8175E-01**
F8	**6.8847E-11**	1.2264E+01	**3.5845E-12**	1.3473E+01
F9	**1.7825E-14**	0.0000E+00	**9.8192E-15**	0.0000E+00
F10	**2.5067E-09**	1.6168E-08	2.3197E-09	**1.4097E-09**
F11	2.6284E-10	**6.7423E-11**	**4.7477E-11**	5.6309E-11
F12	**2.5738E-13**	5.2260E-13	**9.7933E-14**	**7.3866E-14**
F13	5.3530E-13	**3.0608E-13**	**4.4706E-13**	**2.6744E-13**
F14	1.4440E-15	**1.2862E-15**	**1.4267E-15**	1.7586E-15
F15	6.1666E-12	**3.4063E-14**	**3.1147E-14**	**1.7152E-14**
F16	1.5544E-14	**6.5956E-15**	**6.1553E-15**	6.6566E-15
F17	**6.0061E-14**	7.3601E-13	**1.5832E-11**	**8.7437E-14**
F18	**1.5891E-13**	1.0284E-12	**6.3152E-14**	1.6779E-09
F19	**6.2486E-15**	9.2926E-03	**5.3559E-15**	**2.0085E-15**
F20	8.2629E-15	**8.1218E-15**	**5.6553E-15**	**5.3160E-15**
F21	1.8562E-12	**1.3714E-12**	**4.6740E-13**	**3.6452E-13**
F22	7.9963E-13	**6.8134E-13**	**9.2293E-14**	3.1330E-13
F23	**1.5963E-13**	3.3840E-13	**9.2293E-14**	7.0228E-13

The best of the comparison results are in [bold].

In Table 12 (D = 300), only for the unimodal functions F1 and F2, the Std values obtained by the LFGOA algorithm are 1.3549E-10 and 1.1144E-64, for the multimodal function F11 and F13, the Std values obtained by the LFGOA algorithm is 6.7423E-11 and 3.0608E-13, for the composite functions F14–F16 and F20–F22, the Std values obtained by the LFGOA algorithm are 1.2862E-15, 3.4063E-14, 6.5956E-15, 8.1218E-15, 1.3714E-12, and 6.8134E-13, which are less than the GOA algorithm.

In Table 12 (D = 300), for the unimodal functions F1, F2, F7, the Std values obtained by the LFGOA algorithm are 3.0527E-11, 1.4144E-82, and 2.8175E-01, for the multimodal function F10, F12, and F13, the Std values obtained by the LFGOA algorithm are 1.4097E-09, 7.3866E-14, and 2.6744E-13, for the composite functions F15, F17 and F19–F21, the Std values obtained by the LFGOA algorithm are 1.7152E-14, 8.7437E-14, 2.0085E-15, 5.3160E-15, and 3.6452E-13, which are less than the GOA algorithm.

**Table 13:** The best values of dimensions are equal to 50 and 100.

	$f_{\textbf {best}} \ (D=50$)		$f_{\textbf {best}} \ (D=100$)	
	GOA	LFGOA	GOA	LFGOA
F1	3.6191E+01	**1.3103E-09**	1.2055E+00	**2.1951E-11**
F2	1.3482E-03	**2.4163E-21**	2.4131E-05	**1.9438E-31**
F3	2.3206E-03	**9.8987E-12**	5.8872E-03	**1.1903E-12**
F4	6.5617E-02	**7.8056E-06**	1.5523E-02	**5.1792E-07**
F5	**1.7916E+00**	3.7966E+00	**3.8084E+00**	3.8440E+00
F6	**3.9447E-03**	3.8284E-01	**3.5299E-04**	2.3066E-01
F7	2.1050E-02	**9.9672E-05**	1.4000E-02	**1.3129E-04**
F8	−1.5817E+03	−1.4125E+03	−1.7396E+03	−1.5779E+03
F9	9.9496E+00	**0.0000E+00**	2.0894E+01	**0.0000E+00**
F10	2.9110E-02	**1.0108E-09**	1.8809E-04	**1.4488E-09**
F11	2.8866E-01	**6.6189E-09**	2.1058E-01	**3.5065E-10**
F12	9.0110E-04	**2.9048E-04**	**7.2328E-05**	9.2275E-05
F13	**2.8781E-04**	3.5316E-03	2.0603E-04	**1.9707E-05**
F14	**9.9800E-01**	**9.9800E-01**	**9.9800E-01**	**9.9800E-01**
F15	1.6420E-03	**2.0722E-03**	**1.6133E-03**	1.3146E-03
F16	**−1.0316E+00**	**−1.0316E+00**	**−1.0316E+00**	**−1.0316E+00**
F17	**3.9789E-01**	**3.9789E-01**	**3.9789E-01**	**3.9789E-01**
F18	**3.0000E+00**	**3.0000E+00**	**3.0000E+00**	**3.0000E+00**
F19	**−3.8556E+00**	−9.9789E-01	**−3.8628E+00**	−3.5906E+00
F20	**−3.3220E+00**	−3.2503E+00	−3.2031E+00	**−3.3220E+00**
F21	−1.0153E+01	**−5.0552E+00**	−1.0153E+01	**−5.0552E+00**
F22	−1.0403E+01	**−5.0877E+00**	−1.0403E+01	**−5.0877E+00**
F23	−5.1756E+00	**−5.1285E+00**	−1.0536E+01	**−5.1285E+00**

The best of the comparison results are in [bold].

In Table 13 (D = 50, D = 100), there are 17 out of 23 best values obtained by the LFGOA algorithm, such that the number is far exceeded by the GOA algorithm.

**Table 14:** The best values of dimensions are equal to 300 and 500.

	$f_{\textbf {best}} \ (D=300$)		$f_{\textbf {best}} \ (D=500$)	
	GOA	LFGOA	GOA	LFGOA
F1	1.3993E-01	**4.8395E-13**	4.4886E-02	**1.0601E-13**
F2	1.0234E-04	**1.7641E-67**	3.8426E-05	**2.0369E-93**
F3	3.4992E-07	**1.2297E-13**	2.8058E-09	**4.8504E-16**
F4	6.1177E-05	**5.2908E-08**	2.2484E-05	**2.5675E-08**
F5	**2.2175E+01**	3.4648E+00	**4.4415E-01**	3.2320E+00
F6	**9.6501E-08**	1.5732E-01	**6.1218E-09**	1.2891E-01
F7	2.5094E-03	**1.6588E-05**	1.7812E-03	**3.3404E-05**
F8	-1.5422E+03	-1.3501E+03	-1.8565E+03	-1.2817E+03
F9	1.1940E+01	**0.0000E+00**	3.9798E+00	**0.0000E+00**
F10	2.8376E-05	**2.1260E-09**	1.3150E-05	**2.3226E-10**
F11	1.0846E-01	**1.8996E-13**	2.9332E-01	**5.6066E-14**
F12	**6.9018E-09**	2.4229E-08	**1.3700E-10**	-9.9993E-01
F13	**5.2347E-09**	1.1367E-08	**2.7928E-09**	**2.5812E-09**
F14	**9.9800E-01**	**9.9800E-01**	**9.9800E-01**	**9.9800E-01**
F15	2.0363E-02	**1.2232E-03**	**1.2232E-03**	**1.2232E-03**
F16	**-1.0316E+00**	**-1.0316E+00**	**-1.0316E+00**	**-1.0316E+00**
F17	**3.9789E-01**	**3.9789E-01**	**3.9795E-01**	**3.9789E-01**
F18	**3.0000E+00**	**3.0000E+00**	**3.0000E+00**	1.9540E+03
F19	**-3.8628E+00**	-1.0008E+00	**-3.8628E+00**	-1.0008E+00
F20	**-3.3220E+00**	**-3.3220E+00**	**-3.3220E+00**	**-3.3220E+00**
F21	-1.0153E+01	**-5.0552E+00**	-1.0153E+01	**-5.0552E+00**
F22	-1.0403E+01	**-5.0877E+00**	**-5.0877E+00**	**-5.0877E+00**
F23	-5.1756E+00	**-5.1285E+00**	-5.0877E+00	**-5.1285E+00**

The best of the comparison results are in [bold].

In Table 14 (D = 300, D = 500), there are 17 out of 23 best values obtained by the LFGOA algorithm, such that the number is far exceeded by the GOA algorithm.

### Some quantitative metrics of LFGOA algorithm

**Figure 7:**
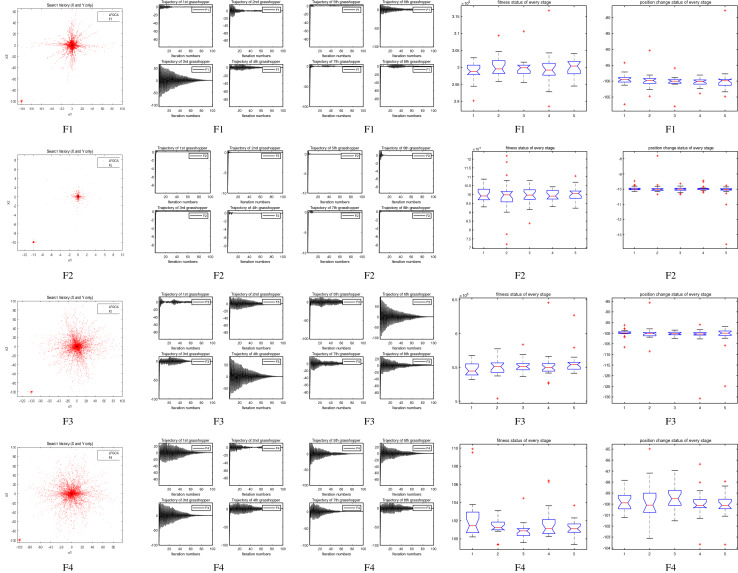

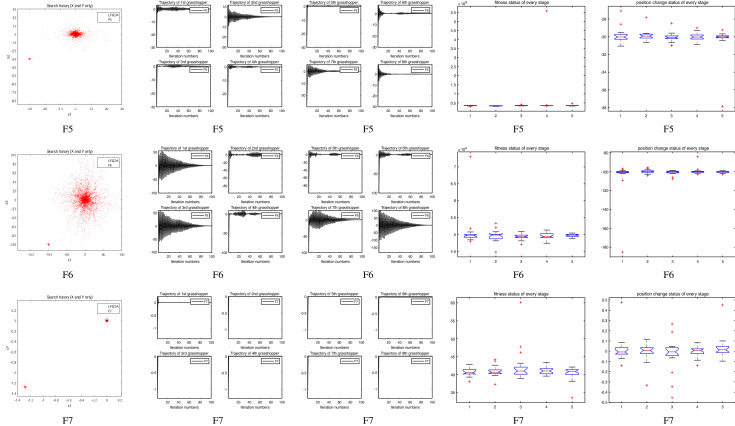
The quantitative metrics about the unimodal test functions F1−F7.

**Figure 8:**
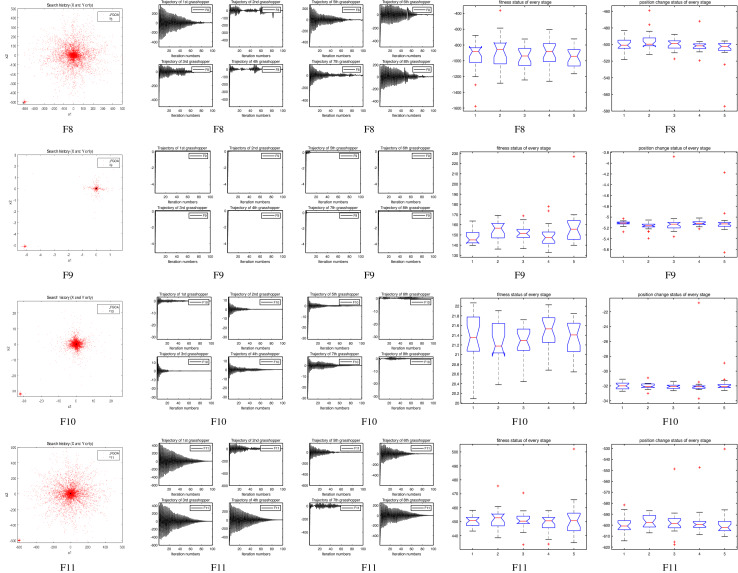

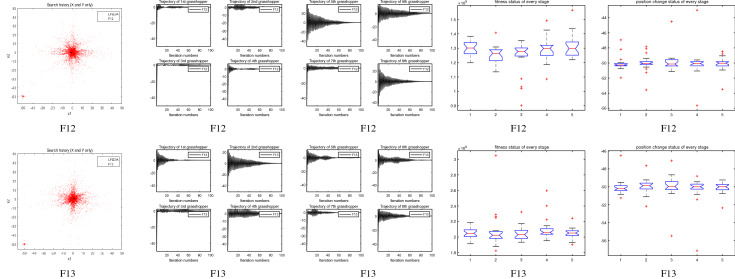
The quantitative metrics about the multimodal test functions F8–F13.

**Figure 9:**
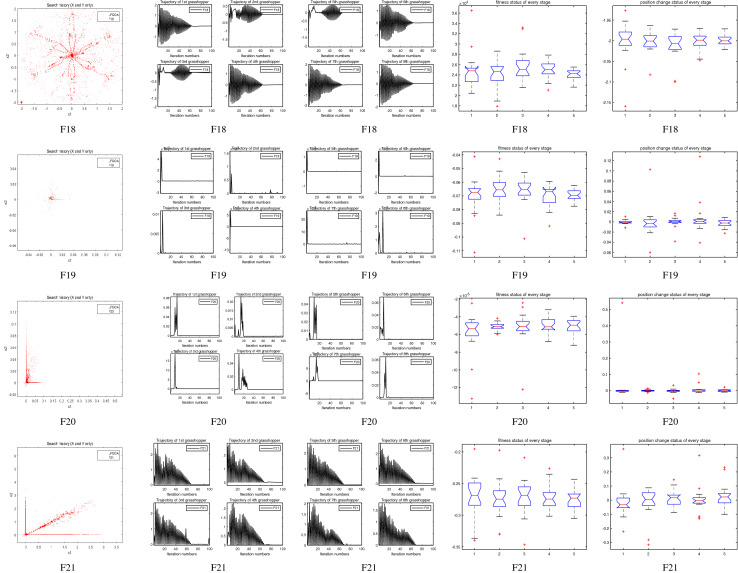

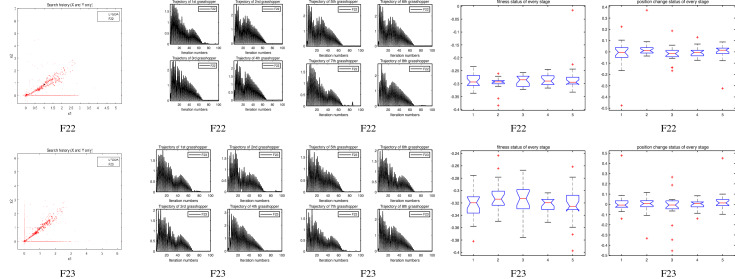
The quantitative metrics about the composite test functions F14–F23.

In the first, second and third columns of Fig. 7, Fig. 8, and Fig. 9, the quantitative metrics about the dynamic change of grasshopper position (search history), and the eight grasshopper trajectories are employed from the first to the last iteration. Tracking the position change of grasshoppers during optimization, we can observe how the LFGOA algorithm explores and exploits the search space. Monitoring eight grasshopper trajectories during optimization, we can know in detail the movements of eight grasshoppers respectively.

From the first column in Fig. 7, we can see that the search history of grasshoppers is mostly concentrated in one region, which indicating that the LFGOA algorithm can quickly search for promising regions. In order to see the changes of the grasshoppers’ positions during searching, the trajectories of eight grasshoppers are picked in the second and third columns in Fig. 7, Fig. 8, and Fig. 9 as well. In the fourth and fifth columns of Fig. 7, the Box plot is used to check affirmed of the LFGOA algorithm stability. In the fourth column of Fig. 7, the Box plot is used to depict the fitness status by five groups (each group covering 20 iterations) at every stage. In the fifth column of Fig. 7, the Box plot is used to depict the position change by five groups (each group covering 20 iterations) at every stage.

#### The unimodal test functions F1–F7

In the first columns in Fig. 7, for the unimodal test functions F1–F7, it can be clearly seen that agents tend to exploration promising regions of the search space and exploitation very accurately around the global optima over the course of iterations in the form of rough like adozens of agents clustered together.

In the second and third columns in Fig. 7, the trajectory graphs of eight grasshoppers (as representative of all grasshoppers) are selected to show the grasshopper’s dynamic position changes respectively during optimization. From the second and the third columns in Fig. 7: we can see that the third in F2 and F7, the fifth and the seventh in F1, the fifth in F5, all of these grasshoppers undergo slight fluctuations during the grasshoppers searching respectively. From the second and third columns of Fig. 7: we also can see trajectory curves that the third in F1, the fourth and the sixth in F3, the third in F4, the first, the third and the eighth in F6, all of the grasshoppers made abrupt largely fluctuations in the initial stages of optimization respectively. Exploration of search space takes place due to high repulsive rate of the LFGOA algorithm. It is also seen that, as these grasshopper’s optimization approaches further the fluctuation decreased gradually over the course of iterations. This is done due to the attraction forces as well as comfort zone between grasshoppers. According to Berg et al.^38^, this behaviour can guarantee that an algorithm eventually convergences to a point and search locally in a search space.

There are some mild autocorrelations and cross-linked between the trajectory graphs of grasshopper in the first columns of Fig. 7 with the second and third columns of Fig. 7, and the search history of grasshoppers in the first column of Fig. 7, the small fluctuation of the grasshoppers corresponding to the small scatter graph of the grasshopper clustered together, the great fluctuation of the grasshoppers corresponding to the big scatter graph of the grasshopper clustered together. It is meaningful on some extent, the inferences about the effectively convergence of the LFGOA algorithm while avoiding most locally optimal from the trajectory graphs of grasshopper and search history of grasshoppers.

To analysis the LFGOA randomness nature, the Box plot is used to show the difference by comparisons the fitness about the LFGOA algorithm. As the box contains 50% of the data, therefore, the height of the box can directly reflect the fluctuation level of the fitness about the LFGOA algorithm. The box plot is relatively short for the unimodal benchmark function F5 in the fourth column of Fig. 7 that reflects the fluctuation of the fitness is slight, which corresponding little promising regions of the search space of F5 in the first column in Fig. 7. There are more or little outliers among the entire unimodal benchmark functions F1–F7, which corresponding there are separate scatter clustered regions, except the big promising regions of the search space. The box plot is relatively tall for the unimodal benchmark function F4 in the fifth column of Fig. 7 that reflects the fluctuation of the position changes are great at every search stage, which corresponding the grasshoppers made abrupt largely fluctuations in the initial stage of optimization respectively in the second and third columns in Fig. 7.

#### The multimodal test functions F8–F13

From the first column in Fig. 8, for the multimodal benchmark functions F8–F13, it can be clearly seen that agents tend to exploration promising regions of the search space and exploitation very accurately around the global optima over the course of iterations in the form of rough like adozens of agents clustered together.

From the second and third columns of Fig. 8: we can see that the F9, the sixth and the eighth in F10, the third in F12, all of the grasshoppers undergo slight fluctuations during the grasshoppers searching respectively. From the second and the third columns in Fig. 8: the first, the fifth, the sixth, the seventh, and the eighth in F8; the first, the third, the fourth, the sixth, and the eighth in F11; the fifth, the sixth, and the eighth in F12; the third in F13; all of these grasshoppers made abrupt largely fluctuations in the initial stages of optimization respectively during the grasshoppers searching respectively.

There is not outlier in the box plot of the multimodal benchmark function F10 in the fourth column of Fig. 8 that reflects the fluctuation of the fitness is not large and the grasshoppers clustered around a relatively little promising regions of the search space.

There are more or little outliers among the entire multimodal benchmark functions F8–F13 in the fifth column of Fig. 8, which corresponding there are separate scatter clustered regions, except the big promising regions of the search space.

#### The composite test functions F14–F23

From the first column in Fig. 9, for the composite benchmark functions F14 and F15, it can be clearly seen that agents tend to exploration promising regions of the search space and exploitation very accurately around the global optima over the course of iterations in the form of rough like adozens of agents clustered together. From the first column in Fig. 9, for the composite benchmark functions F21, F22 and F23, from a search history point of view, the agents tend to extensively exploration promising regions of the search spaces and exploitation the best target in the form of the scatter shape is rough like a thin stripe shape.

From the second and third columns of Fig. 9, we can see that the F21, F22, and F23, all of these grasshoppers made abrupt largely fluctuations from positive to the zero with one direction in the initial stage of optimization respectively during the grasshoppers extensively searching. There are more or little outliers among the composite benchmark function in the fourth column of Fig. 9, which corresponding there are separate scatter clustered regions, except the big promising regions of the search space. There are more or little outliers among the entire composite benchmark functions in the fifth column of Fig. 9, which corresponding there are separate scatter clustered regions, except the big promising regions of the search space.

### Computational complexity of the LFGOA

In this section, the general computational complexity of the LFGOA is presented. The computational complexity of the LFGOA typically relies on three rules: solutions initialization, calculate the fitness functions, and updating of solutions. In the associated formulas, *N* indicates the number of individuals in the population (the number of solutions), and *T* represents the maximum quantity of iterations. During the initial stage, the computational complexity of fitness evaluation is *O*(*N*). The computational complexity of the solutions’ updating processes is $O (T \times N) + O (T \times N\times Dim)$, which consists of exploring for the best positions and updating the solutions’ positions of all solutions, where the dimension size of the given problem is called Dim. From the above analysis, we can acquire the total computational complexity of the LFGOA is $O (N \times (T \times Dim+1))$.

## Results and discussion

As we can see in Section 4, the LFGOA algorithm significantly outperforms others in terms of numerical optimization. There are several reasons why the LFGOA algorithm did perform well in most of the test cases. First, Levy-flight strategy: Levy flight can increase the diversity of the population and make the algorithm jump out of local optimum more effectively. This approach is helpful to make LFGOA faster and more robust than GOA. Second, in GOA, it is assumed that the fittest grasshopper (the one with the best objective value) during optimisation is the target. This will assist GOA to save the most promising target in the search space in each iteration and requires grasshoppers to move towards it. This is done with the hope of finding a better and more accurate target as the best approximation for the real global optimum in the search space.

Therefore, this approach promotes the exploration of promising feasible regions and is the main reason for the superiority of the LFGOA algorithm. Third, the LFGOA algorithm has an explicit restart mechanism. These are the reasons why LFGOA performs better than other algorithms at the end of the results section. Another finding in the results is the performance of most of the AHA, AO, DA, DMOA, GBO, HGS, HHO, and MVO are not good enough. There is no restart mechanism for significant abrupt movements in the search space and this is likely to be the reason the performance of most of the eight algorithms is not good enough. In summary, the discussion and findings of this work clearly demonstrate the quality of the exploration, exploitation, local optima avoidance, and convergence rate of the LFGOA algorithm.

### Real application of LFGOA in constrained engineering problems

Engineering constrained optimization problems are complex, sometimes even the optimal solutions of interest do not exist^39^. Engineering constrained optimization problems have been utilized by many researchers to evaluate the performance of different algorithms^40^. Although the above-discussed results prove and verify the high performance of the LFGOA algorithm, there is also to confidently confirm the performance of this algorithm in engineering constrained optimization problems in real life. In this section, the effectiveness of the LFGOA algorithm is verified in terms of its ability to solve constrained engineering optimization problems in practical application; seven well-studied constrained engineering design examples are selected to verify the proposed LFGOA algorithm, including: Himmelblau’s nonlinear optimization problem, Cantilever beam design, Car Side Impact Design, Gear train Design, Pressure vessel design, Speed Reducer Design, and tabular column design.

However, different real-world problems often have different constraints, so a suitable approach is demanded to deal with such problems^41^. The main idea is to transform the actual optimization problem into a mathematical model, and then use the LFGOA algorithm to find the optimal solution. Normally, *f*(*x*) is the fitness function, *x* represents the search space, $x_1,x_2,\ldots ,x_n$ represent different dimensions, there are several equality and inequality constraints in engineering constrained optimization problems. In order to be suitable for these engineering constrained problems, the search agent of our proposed LFGOA algorithm does not only rely on fitness functions to update the location. So, the simplest method of dealing with constraints (penalty functions) can be used effectively to deal with constraints in algorithms^42^. That is, if the search agent violates any constraints, it will be assigned a large objective function value. This way, it is automatically replaced by a new search agent after the next iteration. So, we use penalty functions in which the LFGOA algorithm has achieved good values if it violates one of these constraints.

### Himmelblau’s nonlinear optimization problem.

Before solving the engineering constrained problems, the LFGOA was benchmarked using a well-known problem, namely, Himmelblau’s problem, which is a relatively complex constrained problem of minimization five positive design variables and six nonlinear inequality constraints, and ten boundary conditions. This problem has originally been proposed by Himmelblau^43^ and it has been widely used as a benchmark nonlinear constrained optimization problem and applied to many fields. The problem can be outlined as follows:

Consider:

$$\begin{aligned} x=\left[ x_1,x_2,x_3,x_4,x_5\right] , \end{aligned}$$

Minimize:

$$\begin{aligned} f\left( x\right) =5.3578547x_3^2+ 0.8356891x_1x_5+ 37.293239x_1 - 40792.141, \end{aligned}$$

Subject to:

$$\begin{aligned}{} & {} {0\le g}_1\left( x\right) \le 92,\\{} & {} {90\le g}_2\left( x\right) \le 110,\\{} & {} {20\le g}_3\left( x\right) \le 25, \end{aligned}$$

Where:

$$\begin{aligned}{} & {} g_1\left( x\right) =85.334407+ 0.0056858x_2x_5+ 0.0006262x_1x_4- 0.0022053x_3x_5\\{} & {} g_2\left( x\right) =80.51249+ 0.0071317x_2x_5+ 0.0029955x_1x_2- 0.0021813x_3^2\\{} & {} g_3\left( x\right) =9.300961+\ 0.0047026x_3x_5+\ 0.0012547x_1x_3+0.0019085x_3x_4\\{} & {} {78\le x}_1\le 102,\\{} & {} {33\le x}_2\le 45,\\{} & {} 27\le x_3,\ x_4,\ x_5\le 45\\ \end{aligned}$$

**Figure 10:**
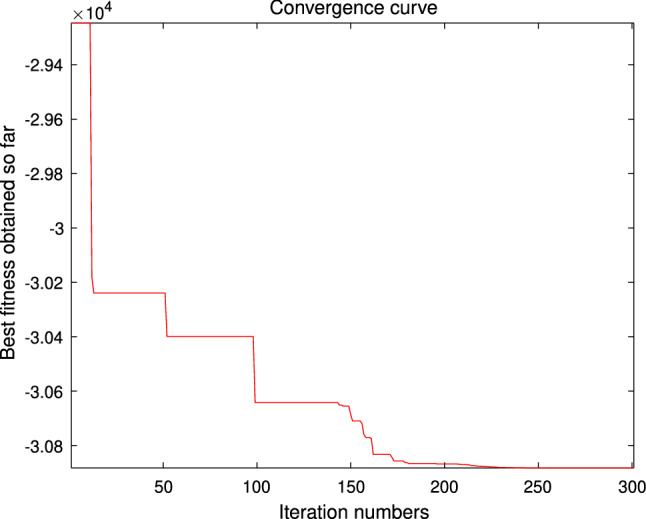
Convergence curve for Himmelblau’s constraint problem.

Table 15 demonstrates the comparison of the best solution among the different optimizers and the corresponding design variables, while the statistical results for each considered strategy are detailed in Table 16. The results obtained by LFGOA algorithm are compared with five state-of-the-art algorithms, such as Artificial Bee Colony algorithm^44^, sparrow search algorithm^45^, Cuckoo search algorithm^46^, harmony search algorithm^47^, and Differential gradient evolution plus algorithm^48^ respectively in the literatures. It can be clearly seen that the LFGOA algorithm performed better without any violation and is feasible on this issue. The convergence curve in Fig. 10 shows the function values versus the iteration numbers for the constrained problem.

**Table 15:** Reported results for constrained problems from different optimizers.

Algorithm	Optimal values for variables					f(x)
	x1	x2	x3	x4	x5	
LFGOA	78.00	34.41	27.76	40.88	44.56	−30850.53
ABC	78.00	33.00	27.07	45.00	44.97	−31025.58
SSA	78.00	33.00	30.00	45.00	36.78	−30665.54
CS	78.00	33.00	30.00	45.00	36.78	**−30665.23**
HS	78.00	33.00	30.00	45.00	36.78	−30665.50
DGE+	78.00	33.00	30.00	45.00	36.78	−30665.54

The best of the comparison results are in [bold].

**Table 16:** Comparative results of LFGOA with other methods for Himmelblau’s (N/A stands for not available).

Algorithm	Best	Mean	Std
LFGOA	−30850.5347	−30859.2667	16.5467
ABC	−31025.5820	**−30665.5390**	**0.0000**
SSA	−30665.5387	−30665.3808	0.5713
CS	**−30665.2327**	N/A	11.6231
HS	−30665.5432	N/A	N/A
DGE+	−30665.5391	−30665.5391	0.0001

The best of the comparison results are in [bold].

### Cantilever beam design

Cantilever beam design is a type of concrete engineering problems. It works to minimize the total weight of a cantilever beam by optimizing the hollow square cross-section parameters. There are five squares of which the first block is fixed and the fifth one burdens a vertical load.

**Figure 11:**
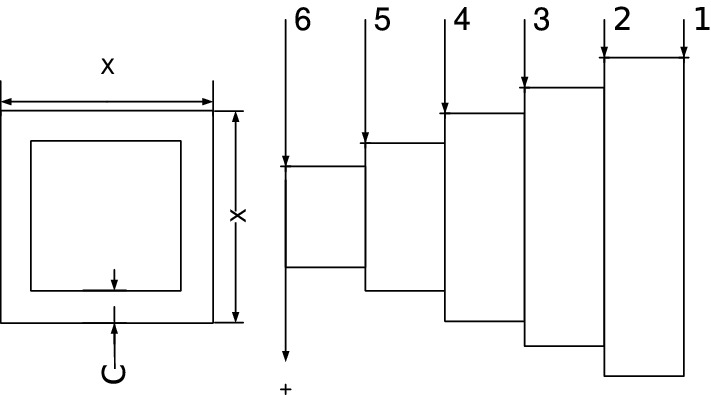
Schematic of cantilever beam.

**Figure 12:**
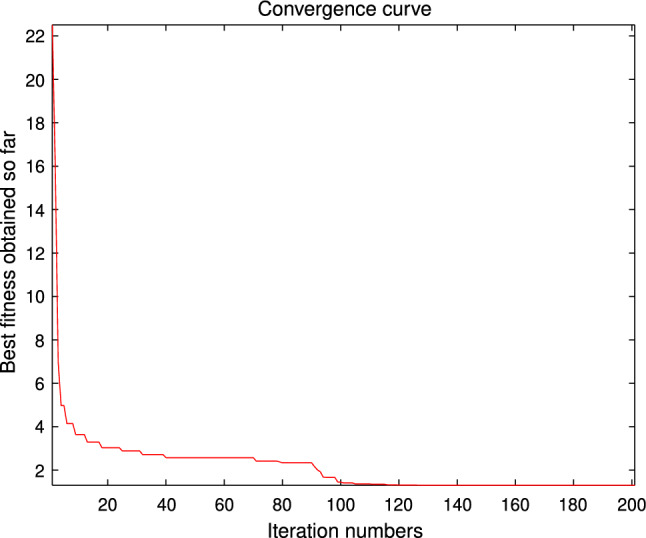
Convergence of cantilever beam.

For this well-known case, Fig. 11 shows the shape of the cantilever beam, the beam is rigidly supported at right side end, and a vertical force acts on the cantilever free node of the left side, which is supported at the rightmost block and the other blocks are left free. The widths and heights of the five beams considered of the problem are used as design parameters of the optimization. The beam consists of five hollow square blocks with constant thickness, whose heights (or widths) are the decision variables. The cantilever weight optimization is formulated in the following equation:

Consider:

$$\begin{aligned} x=\left[ x_1,x_2,x_3,x_4,x_5\right] , \end{aligned}$$

Mathematically speaking, it is possible to write most optimization problems in the generic form:

Minimize:

$$\begin{aligned} f\left( x\right) =0.06224\left( x_1+x_2+x_3+x_4+x_5\right) , \end{aligned}$$

Subject to:

$$\begin{aligned} g\left( x\right) = \frac{61}{x_1^3}+\frac{27}{x_2^3}+\frac{19}{x_3^3}+\frac{7}{x_4^3}+\frac{1}{x_5^3}-1\le 0, \end{aligned}$$

Variable range:

$$\begin{aligned} 0.01\le x_1,x_2,x_3,x_4,x_5\le 100. \end{aligned}$$

To evaluate the performance of the proposed LFGOA in solving this problem, some of the algorithms that are chosen for comparison are Artificial hummingbird algorithm^29^ and Gradient-Based Optimizer^33^ in the literatures. The results obtained by LFGOA and their comparison with the aforementioned state-of-the-art metaheuristics are reported in Tables 17 and 18, while the statistical results for each considered strategy are detailed in Table 18. From Tables 17 and 18, it can be seen that LFGOA achieves the high-quality solution for this case. The results of LFGOA algorithm for this problem are consistent to those of other real problems, in which the LFGOA algorithm outperforms the other two algorithms and is the first most efficient approach, and shows very competitive results. The comparative results show that our method can effectively solve this case and reveal better design.

**Table 17:** Results of the comparative algorithms for solving the cantilever beam design problem.

Algorithm	Optimal values for variables					f(x)
	x1	x2	x3	x4	x5	
LFGOA	6.008900	5.304900	4.502300	3.507700	2.150400	**1.336554**
AHA	6.013830	5.302425	4.496347	3.508429	2.152705	1.339965
GBO	6.012400	5.312900	4.494100	3.503600	2.150600	1.339957

The best of the comparison results are in [bold].

**Table 18:** Comparative results of LFGOA with other methods for cantilever beam design.

Algorithm	Best	Mean	Std
LFGOA	**1.3366**	**1.3377**	0.0016
AHA	1.3400	1.3401	**0.0000**
GBO	1.3400	1.3400	**0.0000**

The best of the comparison results are in [bold].

It is evident from Tables 17 and 18 that the proposed LFGOA algorithm performed better without any violation. The convergence curve shows the function values versus the Iteration numbers for the constrained problem are given in Fig. 12.

### Car side impact design

On the foundation of the European Enhanced Vehicle-Safety Committee (EEVC) procedures, a car is exposed to a side impact, and the aim of this benchmark problem is minimizing the weight of the door. There are eleven influence parameters in this problem, which describe as follow:

- the thicknesses of B-pillar inner $(x_1)$,
- the B-pillar reinforcement $(x_2)$,
- the floor side inner $(x_3)$,
- the cross members $(x_4)$,
- the door beam $(x_5)$,
- the door beltline reinforcement $(x_6)$,
- the roof rail $(x_7)$,
- the materials of B-pillar inner $(x_8)$,
- the floor side inner $(x_9)$,
- the barrier height $(x_{10})$,
- the hitting position $(x_{11}).$

Consider:

$$\begin{aligned} x=\left[ x_1,x_2,x_3,x_4,x_5,x_6,x_7,x_8,x_9,x_{10},x_{11}\right] , \end{aligned}$$

Structural weight and response to impact can be approximated using global response surface methodology in order to simplify the analytical formulation of the optimization problem and speed up computations. As an optimization problem, mathematically speaking, it is possible to write simplified models optimization problems in the generic form:

Minimize:

$$\begin{aligned} f\left( x\right) =1.98+4.90x_1+6.67x_2+6.98x_3+4.01x_4+1.78x_5+2.73x_7 \end{aligned}$$

Ten constraints are imposed on the design problem.

Subject to:

$$\begin{aligned}{} & {} g_1\left( x\right) =1.16-0.3717x_2x_4-0.00931x_2x_{10}-0.484x_3x_9+0.01343x_6x_{10}-1 \le 0,\\{} & {} g_2\left( x\right) =46.36-9.9x_2-12.9x_1x_2 + 0.1107x_3x_{10}-32 \le 0,\\{} & {} g_3\left( x\right) =33.86+2.95x_3+0.1792x_1-5.057x_1x_2-11.0x_2x_8-0.0215x_5x_{10}-9.98x_7x_8+22.0x_8x_9-32 \le 0,\\{} & {} g_4\left( x\right) =28.98+3.818x_3-4.2x_1x_2+0.0207x_5x_{10}+6.63x_6x_9-7.7x_7x_8+0.32x_9x_{10}-32 \le 0,\\{} & {} g_5\left( x\right) =0.261-0.0159x_1x_2-0.188x_1x_8-0.019x_2x_7+0.0144x_3x_5+0.0008757x_5x_{10}+0.08045x_6x_9\\{} & {} \quad +0.00139x_8x_{11}+ 0.00001575x_{10}x_{11}-0.32 \le 0,\\{} & {} g_6\left( x\right) =0.214+0.00817x_5-0.131x_1x_8-0.0704x_1x_9+0.03099x_2x_6-0.018x_2x_7+0.0208x_3x_8\\{} & {} \quad + 0.121x_3x_9-0.00364x_5x_6+0.0007715x_5x_{10}-0.0005354x_6x_{10}+0.00121x_8x_{11}\\{} & {} \quad +0.00184x_9x_{10}-0.02x_2^2-0.32 \le 0,\\{} & {} g_7\left( x\right) =0.74-0.61x_2-0.163x_3x_8 + 0.001232x_3x_{10}-0.166x_7x_9 + 0.227x_2^2-0.32 \le 0,\\{} & {} g_8\left( x\right) =4.72-0.5x_4-0.19x_2x_3- 0.0122x_4x_{10}+ 0.009325x_6x_{10}+ 0.000191x_{11}^2-4 \le 0,\\{} & {} g_9\left( x\right) =10.58-0.674x_1x_2-1.95x_2x_8 + 0.02054x_3x_{10}-0.0198x_4x_{10}+ 0.028x_6x_{10}-9.9 \le 0,\\{} & {} g_{10}\left( x\right) =16.45-0.489x_3x_7-0.843x_5x_6 + 0.0432x_9x_{10}-0.0556x_9x_{11}- 0.000786x_{11}^2-15.7 \le 0,\\ \end{aligned}$$

The simple bounds of this problem are:

$$\begin{aligned}{} & {} 0.5\le x_1,x_3,x_4\le 1.5,\\{} & {} 0.45{\le x}_2\le 1.35,\\{} & {} 0.875\le x_5\le 2.625\\{} & {} 0.4\le x_6,x_7\le 1.2\\{} & {} 0.192\le x_8,x_9\le 0.345\\{} & {} -30\le x_{10},x_{11}\le + 30. \end{aligned}$$

To evaluate the performance of the proposed LFGOA algorithm in solving this problem, some of the algorithms that are chosen for comparison are Social Network Search^49^, Enhanced grasshopper optimization algorithm^19^, and Firefly Algorithm^50^ respectively in the literatures.

The results obtained by LFGOA and their comparison with the aforementioned state-of-the-art metaheuristics are reported in Table 19, while the statistical results for each considered strategy are detailed in Table 20.

**Table 19:** The optimum values of the car side impact design example.

	LFGOA	SNS	EOBL-GOA	FA
x1	0.5082	0.5000	0.5000	0.5000
x2	0.8902	1.1159	1.1164	1.3600
x3	0.5000	0.5000	0.5000	0.5000
x4	1.2635	1.3029	1.3021	1.2020
x5	0.5000	0.5000	0.5000	0.5000
x6	1.0048	1.5000	1.5000	1.1200
x7	0.5000	0.5000	0.5000	0.5000
x8	0.3233	0.3450	0.3450	0.3450
x9	0.2810	0.1920	0.1920	0.1920
x10	5.2244	−19.6389	−19.5494	8.8731
x11	12.0882	0.0000	−0.00431	−18.9981
f(x)	21.2196	22.8430	22.8429	22.8430

**Table 20:** Comparative results of LFGOA with other methods for car side impact design.

Algorithm	Best	Mean	Std
LFGOA	**21.2196**	22.9125	0.0417
SNS	22.8430	22.8815	0.1018
EOBL-GOA	22.8430	**22.8351**	**0.0243**
FA	22.8430	22.8938	0.1667

The best of the comparison results are in [bold].

**Figure 13:**
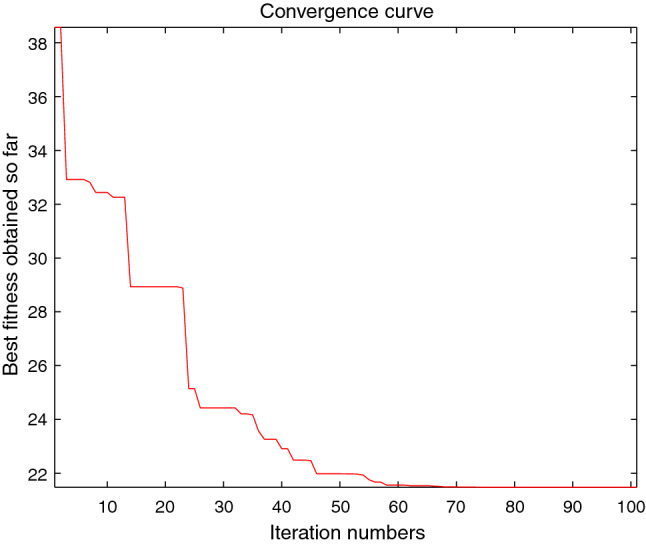
Convergence curve for car side impact design.

It is evident from Tables 19 and 20 that the proposed LFGOA algorithm performed better without any violation. The convergence curve shows the function values versus the Iteration numbers for the constrained problem are given in Fig. 13.

### Discrete engineering problem-gear train design

The high-speed train drive wheel transmission system mostly adopts a gear transmission structure. Due to the limited size of the structure, the pinion gear and the motor drive shaft are connected by an interference fit. The vibration is caused by an unreasonable design, which causes a system failure. The objective of gear train design problem is to minimize the cost of the “Gear ratio” of the gear train in field mechanical engineering problem. The “Gear ratio” defined as the ratio of the angular velocity of the output shaft to the angular velocity of the input shaft, the “Gear ratio” is calculated as follows:

$$\begin{aligned} Gear ratio =\ \frac{angular\ velocity\ of\ output\ shaft}{angular\ velocity\ of\ input\ shaft} \end{aligned}$$

The parameters of this problem are discrete with the increment size of 1 since they define the teeth of the gears $(T_a,T_b,T_c,T_d).$ There constraints are only limited the variable ranges. The design of gear train is a kind of mixed problems which have to determine various types of design variables such as continuous, discrete, and integer variables. This problem simply stated is: given a fix input drive and a number of fixed output drive spindles, how can the spindles be driven by the input using the minimum number of connecting gear in the train. To handle discrete parameters, each search agent was rounded to the nearest integer number before the fitness evaluation.

**Figure 14:**
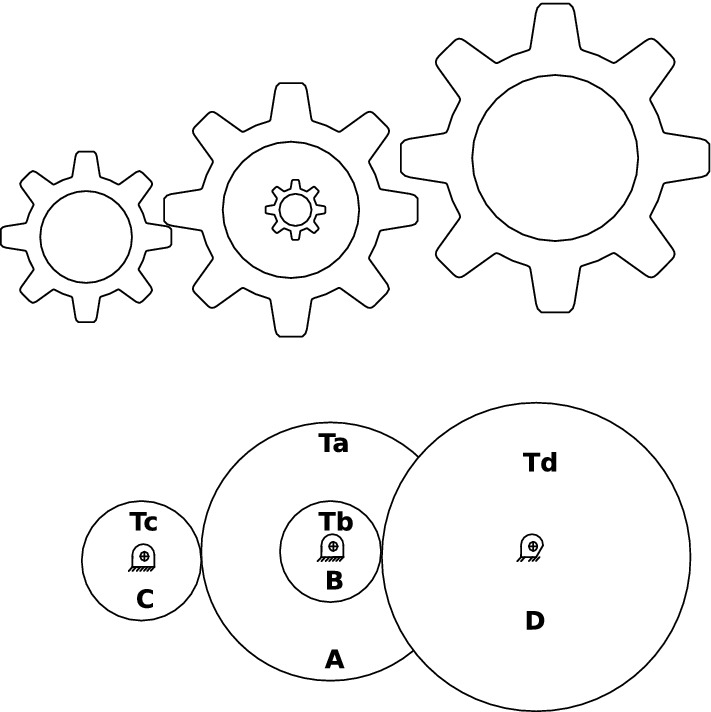
Schematic of gear train.

The number of teeth of gears $T_a(=x_1)$, $T_b(=x_2)$, $T_c(=x_3)$, and $T_d(=x_4)$ are considered as the design variables, and illustrates at Fig. 14.

Consider:

$$\begin{aligned}{}[x_1,x_2,x_3,x_4] = [T_a,T_b,T_c,T_d], \end{aligned}$$

The mathematical formulation is provided as follows:

Minimize:

$$\begin{aligned} f\left( x\right) ={(\frac{1}{6.931}-\frac{x_3x_2}{x_1x_4})}^2 \end{aligned}$$

The design engineering constraint is defined as the number of teeth on any gear that should only be in the range of [12, 60], in other words, the constraints are only limited the variable ranges: $12 \le {\ x}_1,x_2,x_3,x_4\ \le 60$

**Figure 15:**
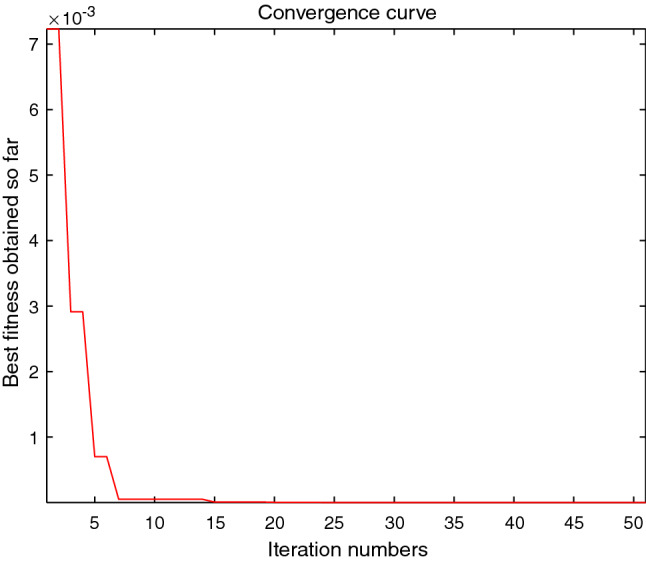
Convergence of gear train.

This section uses the proposed LFGOA algorithm to solve the gear train design problem and compares the results with other optimization algorithms, including Social Network Search^49^, An enhanced hybrid arithmetic optimization algorithm^51^, The Ant Lion Optimizer^52^, and Multi-Verse-Optimizer^36^ respectively in the literatures. Table 21 compares the minimum cost and design variables obtained using the LFGOA algorithm and other optimization algorithms, while the statistical results for each considered strategy are detailed in Table 22.

**Table 21:** Comparison results of the gear train design problem.

Algorithm	Optimal values for variables				Optimal gear ratio
	x1	x2	x3	x4	
LFGOA	32	12	23	59	**2.62E-16**
SNS	43	19	16	49	2.70E-12
CSOAOA	22	16	48	50	2.10E-10
ALO	49	19	16	43	2.70E-12
MVO	43	16	19	49	2.70E-12

The best of the comparison results are in [bold].

**Table 22:** Comparative results of LFGOA with other methods for gear train design.

Algorithm	Best	Mean	Std
LFGOA	**2.62E-16**	**1.35E-16**	**1.30E-16**
SNS	2.70E-12	1.68E-10	3.75E-10
CSOAOA	2.10E-10	2.10E-10	3.82E-10
ALO	2.70E-12	4.72E-09	6.08E-09
MVO	2.70E-12	7.59E-10	1.08E-09

The best of the comparison results are in [bold].

However, the optimal values for variables obtained are different. It is worth pointing out that any feasible solution is an optimal solution, the values in Table 21 which gained by the five algorithms, only rough agreed with each other. Therefore, this design can be considered as a new design with a similar optimal “Gear ratio”. Table 21 shows that the LFGOA algorithm gives competitive results for numbers of function evaluations and is suitable to solve discrete constrained problems. Once more, these results prove that the proposed LFGOA algorithm can solve discrete real problems efficiently. As shown in the Fig. 15, the convergence curve is quickly and the solutions were obtained instantly under satisfy all constraints.

### Pressure vessel design

**Figure 16:**
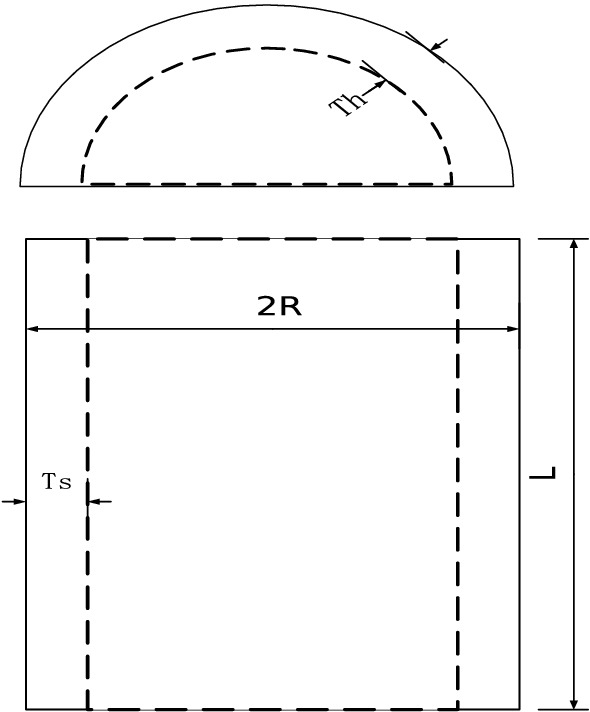
Schematic of pressure vessel.

The pressure vessel design optimization task has also been popular among researchers and optimized in various studies. Pressure vessel design is a mixed discrete-continuous constrained optimization problem. Using rolled steel plate, the shell is made in two halves that are joined by two longitudinal welds to forms a cylinder. The objective of this problem is to minimize the total cost consisting of material, forming, and welding of a cylindrical vessel as in Fig. 16. Both ends of the vessel are capped, and the head has a hemi-spherical shape. There are four variables in this problem:

- Thickness of the shell $(T_s)$,
- Thickness of the head $(T_h)$,
- Inner radius (*R*),
- Length of the cylindrical section without considering the head (*L*).

In pressure vessel, the thickness of the shell $(T_s)$ and head $(T_h)$, the internal radius (*R*), and the extent of the section, minus the head (*L*), are variables to be optimized. This problem is subject to four constraints: $T_s$ and $T_h$ are the available thicknesses of rolled steel plates, which are integer multiples of 0.0625 inch, and *R* and *L* are continuous variables. Many meta-heuristic methods that have been adopted to optimize this problem includes Social Network Search^49^, Composite Differential Evolution with Modified Oracle Penalty Method^53^, Artificial hummingbird algorithm^29^, Manta ray foraging optimization^54^, a Hybrid Co-evolutionary Particle Swarm Optimization Algorithm^55^, the Automatic Dynamic Penalisation method (ADP) for handling constraints with genetic algorithms^56^, and a Hybrid Generalized Reduced Gradient-Based Particle Swarm Optimizer^57^ respectively in the literatures.

**Figure 17:**
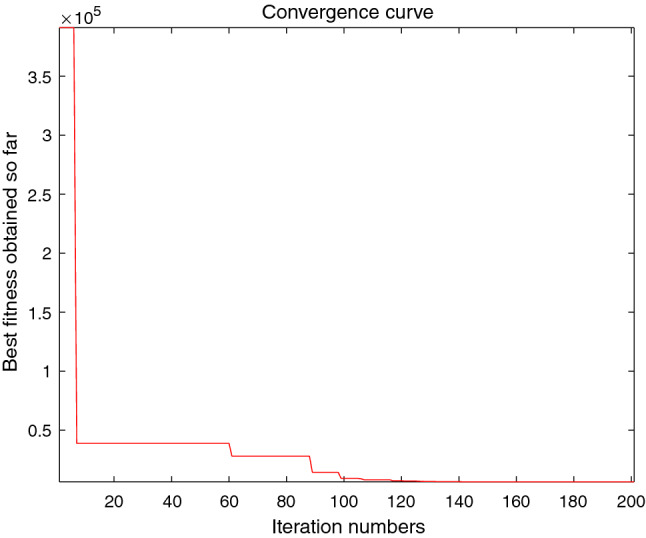
Convergence of pressure vessel.

These constraints and the problem are formulated as follows:

Consider:

$$\begin{aligned} x= (x_1, x_2, x_3, x_4) = (T_s, T_h, R, L), \end{aligned}$$

Minimize:

$$\begin{aligned} f\left( x\right) =0.6224x_1x_3x_4+1.7781x_2x_3^2+3.1661x_1^2x_4+19.84x_1^2x_3, \end{aligned}$$

Subject to:

$$\begin{aligned}{} & {} g_1\left( x\right) =-x_1+0.0193x_3 \le 0,\\{} & {} g_2\left( x\right) =-x_3+0.00954x_3 \le 0,\\{} & {} g_3\left( x\right) =-\pi x_3^2x_4-\frac{4}{3}\pi x_3^3+1296000 \le 0,\\{} & {} g_4\left( x\right) =x_4-240 \le 0, \end{aligned}$$

Variable range:

$$\begin{aligned}{} & {} 0 \le x_1 \le 99,\\{} & {} 0 \le x_2 \le 99,\\{} & {} 10 \le x_3 \le 200,\\{} & {} 10 \le x_4 \le 200, \end{aligned}$$

**Table 23:** Comparison of the best solution for pressure vessel design found by different methods.

Algorithm	Optimal values for variables				f(x)
	x1	x2	x3	x4	
LFGOA	0.7840	0.3875	40.6220	195.8397	5895.5481
SNS	0.8125	0.4375	42.0985	176.6366	6059.7143
MOCoDE	0.8125	0.4375	42.0984	176.6366	6059.7143
AHA	0.7782	0.3847	40.3197	199.9993	**5885.3537**
MRFO	0.7787	0.3849	40.3447	199.6516	5987.8131
ABC	0.7782	0.3847	40.3211	199.9802	5885.4033
CPSOSA	0.8125	0.4375	42.0984	176.6366	6059.7143
ADP_GA	0.8125	0.4375	42.0968	176.6580	6059.9384
PSO_GRG	0.8125	0.4375	42.0984	176.6366	6059.7144
SO	0.7819	0.3857	40.5752	196.5499	5887.5298

**Table 24:** Comparative results of LFGOA with other methods for gear train design.

Algorithm	Best	Mean	Std
LFGOA	5895.5481	5990.7402	65.6885
SNS	6059.7143	6097.1003	92.8000
MOCoDE	6059.7143	6059.7143	**0.0000**
AHA	**5885.3537**	**5885.5382**	0.1378
MRFO	5987.8131	6167.4900	12.6209
CPSOSA	6059.7143	6059.7143	**0.0000**
ADP_GA	6059.9384	6182.0022	122.3256
PSO_GRG	6059.7144	6369.4767	454.8344
SO	5887.5298	5989.8092	104.0000

The best of the comparison results are in [bold].

From Tables 23 and 24, it is evident that LFGOA obtain the better solution among these compared approaches. From Table 24, once more, the statistical results of different methods also demonstrate that the proposed LFGOA method can solve this constrained optimization problems with discrete-continuous variables effectively and provide competitive statistical results. It should be noted the results of LFGOA do not denote that it can find better solutions due to the accuracy.

As shown in the Fig. 17, the convergence curve quickly converge towards the global optimum and the solutions was obtained instantly under satisfy all constraints.

### Speed reducer design

**Figure 18:**
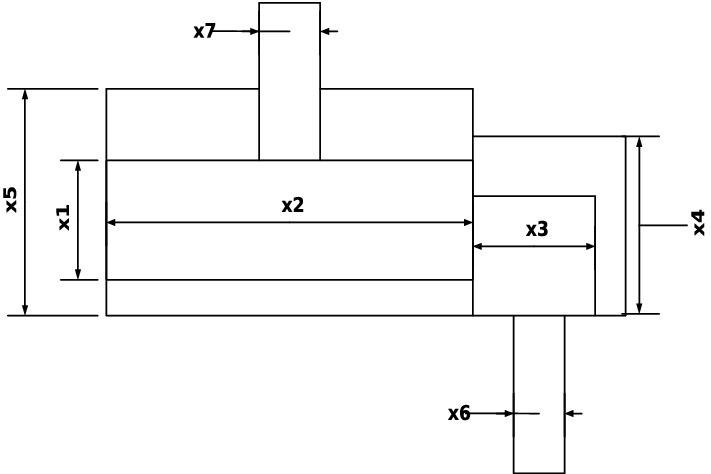
Schematic of speed reducer.

In mechanical systems, one of the essential parts of the gearbox is the speed reducer, and it can be considered as a challenging benchmark engineering problem and can be employed for several applications. In this optimization problem, the weight of the speed reducer is to be minimized with subject to 11 constraints, as shown in Fig. 18. The goal of the speed reducer design problem is to minimize the total weight of the reducer by optimizing the seven variables, which describe as follow:

- the width of the gear surface (cm) $(x_1=b)$,
- the module of teeth (cm) $(x_2=m)$,
- the number of teeth in the pinion $(x_3=p)$,
- the length of the first shaft between bearings (cm) $(x_4=l_1)$,
- the length of the second shaft between bearings (cm) $(x_5=l_2)$,
- the diameter of first shafts (cm) $(x_6=d_1)$,
- the diameter of second shafts (cm) $(x_7=d_2).$

**Figure 19:**
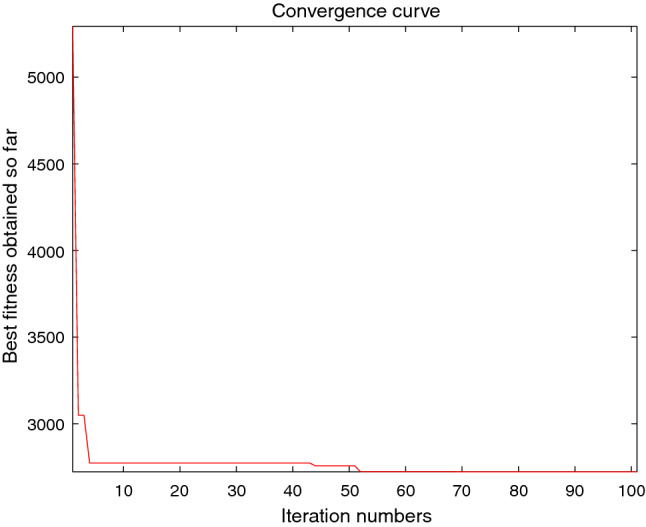
Convergence of speed reducer.

The mathematical model of the gear train design problem is:

Consider variable:

$$\begin{aligned} x = (x_1, x_2, x_3, x_4, x_5, x_6, x_7) = (b, m, p, l_1,\ l_2,\ d_1,\ d_2). \end{aligned}$$

Minimize:

$$\begin{aligned} f\left( x\right)= & {} 0.7854x_1x_2^2\left( 3.3333x_3^2+14.9334x_3-43.0934\right) -1.508x_1\left( x_6^2+x_7^2\right) +7.4777 \left( x_6^3+x_7^3\right) \\{} & {} \quad +0.7854\left( x_4x_6^2+x_5x_7^2\right) , \end{aligned}$$

Subject to:

$$\begin{aligned} g_1\left( x\right)= & {} \frac{27}{x_1x_2^2x_3}-1\le 0,\\ g_2\left( x\right)= & {} \frac{397.5}{x_1x_2^2x_3^2}-1\le 0,\\ g_3\left( x\right)= & {} \frac{1.93x_4^3}{x_2x_6^4x_3}-1\le 0,\\ g_4\left( x\right)= & {} \frac{1.93x_5^3}{x_2x_7^4x_3}-1\le 0,\\ g_5\left( x\right)= & {} \frac{\sqrt{{(\frac{745x_4}{x_2x_3} )}^2+16.9\times {10}^6}}{110x_6^3}-1\le 0\\ g_5\left( x\right)= & {} \frac{\sqrt{{(\frac{745x_4}{x_2x_3} )}^2+157.5\times {10}^6}}{85x_7^3}-1\le 0\\ g_7\left( x\right)= & {} \frac{x_2x_3}{40}-1\le 0,\\ g_8\left( x\right)= & {} \frac{{5x}_2}{x_1}-1\le 0,\\ g_9\left( x\right)= & {} \frac{x_1}{12x_2}-1\le 0,\\ g_{10}\left( x\right)= & {} \frac{1.5x_6+1.9}{x_4}-1\le 0,\\ g_{11}\left( x\right)= & {} \frac{1.1x_7+1.9}{x_5}-1\le 0, \end{aligned}$$

Variable range:

$$\begin{aligned}{} & {} 2.6 \le x_1 \le 3.6,\\{} & {} 0.7 \le x_2 \le 0.8,\\{} & {} x_3\in \{17, 18, 19, . . . , 28 \},\\{} & {} 7.3 \le x_4,\\{} & {} x_5 \le 8.3,\\{} & {} 2.9 \le x_6 \le 3.9,\\{} & {} 5 \le x_7 \le 5.5. \end{aligned}$$

**Table 25:** Results of LFGOA and competitive algorithms in solving the speed reducer design.

Algorithm	Optimal values for variables							f(x)
	x1	x2	x3	x4	x5	x6	x7	
LFGOA	3.5000	0.7000	17.0000	7.4147	7.6669	2.9087	5.0000	**2743.1379**
SNS	3.5000	0.7000	17.0000	7.3000	7.7153	3.3502	5.2867	2994.4711
IDSE	3.6000	0.7000	17.0000	7.3000	8.3000	3.3846	5.5000	3197.8394
CSOAOA	3.5000	0.7000	17.0000	7.3000	7.8000	3.3500	5.2900	2996.3017
AHA	3.5000	0.7000	17.0000	7.3000	7.7153	3.3502	5.2867	2994.4712
MRFO	3.5000	0.7000	17.0000	7.3000	7.7153	3.3502	5.2867	2994.4711
SSA	3.5001	0.7000	17.0000	7.3000	7.8000	3.3512	5.2868	2996.0217
NeGPE-s	3.5000	0.7000	17.0000	7.3000	7.7153	3.3502	5.2867	2990.0000
GBO	3.4999	0.7000	17.0000	7.3000	7.8000	3.3502	5.2866	2996.3481
SO	3.4976	0.7000	17.0000	7.3000	7.8000	3.3501	5.2857	2995.5424

The best of the comparison results are in [bold].

**Table 26:** Comparative results of LFGOA with other methods for speed reducer design.

Algorithm	Best	Mean	Std
LFGOA	**2743.1379**	**2744.1172**	2.3762
SNS	2994.4711	2994.4711	**0.0000**
IDSE	3197.8394	3372.4083	101.3525
CSOAOA	2996.3017	2997.7746	3.5937
AHA	2994.4712	2994.4717	42512.0000
MRFO	2994.4711	2994.4711	0.0146
SSA	2996.0217	3005.5744	4.6300
NeGPE-s	2990.0000	2990.0000	0.0014
GBO	2996.3481	2996.3481	**0.0000**
SO	2995.5424	2995.5424	**0.0000**

The best of the comparison results are in [bold].

This case was previously tackled by many scholars using various heuristic methods, including Social Network Search^49^, Information-Decision Searching Algorithm^58^, An enhanced hybrid arithmetic optimization algorithm^51^, Artificial hummingbird algorithm^29^, Manta ray foraging optimization^54^, sparrow search algorithm^45^, A simplified non-equidistant grey prediction evolution algorithm^59^, Gradient-based optimizer^33^, and Snake Optimizer^5^.

The statistical results of LFGOA and nine optimization methods are compared in Tables 25 and 26. Among the compared optimization algorithms, the LFGOA ranks first as superior to other approaches in optimizing the reducer design, our method can find better geometric variables for this case. Hence, our result is feasible and verifies the effectiveness of the proposed LFGOA algorithm. The results demonstrate that the proposed LFGOA can provide reliable and very comprising solutions compared with the other algorithms.

As shown in the Fig. 19, the convergence curve quickly converge towards the global optimum and the solutions was obtained instantly under satisfy all constraints.

### Tubular column design

**Figure 20:**
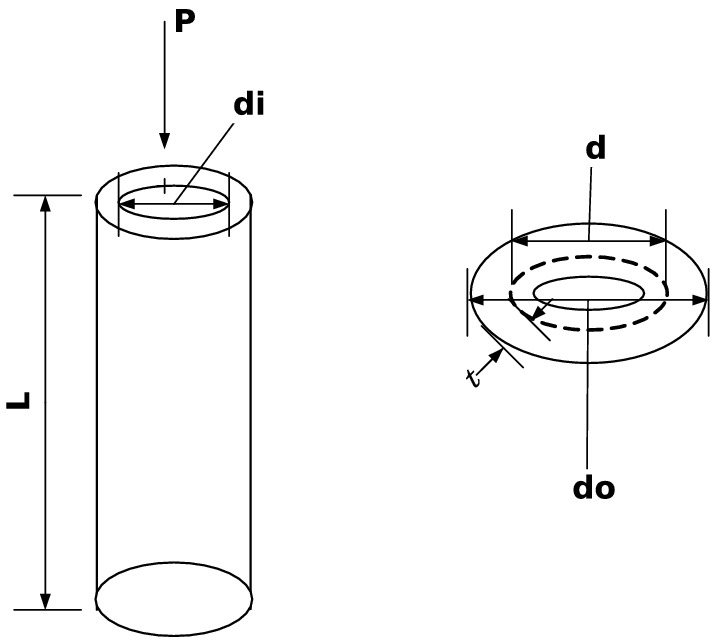
Schematic of tubular column.

Tubular column design is an example of designing a uniform column of the tubular section to carry a compressive load at minimum cost as described in Fig. 20. There are two design variables in this problem, which describe as follow:

- the mean diameter of the column $d (=x_1) (cm)$,
- the thickness of tube $t (=x_2) (cm)$.

The five characteristic parameters in the constituent materials of the column are set as:

- *P* is a compressive load$(= 2500 kgf)$,
- $\sigma _y$ represents the yield stress$(=500 kgf/cm^2)$,
- *E* is the modulus of elasticity$(=0.85\times {10}^6 kgf/cm^2)$,
- $\rho$ is the density$(=0.0025 kgf/cm^3)$,
- *L* denotes the length of the designed column $(= 250 cm)$.

The optimization model of this problem is given as follows:

Consider: $$x=\left[ x_1,x_2\right] =[d,\ \ t],$$

Minimize:

$$\begin{aligned} f\left( x\right) =9.8x_1x_2+2x_1 \end{aligned}$$

Subject to:

$$\begin{aligned} g_1\left( x\right)= & {} \frac{P}{\pi x_1x_2\sigma _y}-1\le 0,\\ g_2\left( x\right)= & {} \frac{8PL^2}{\pi ^3Ex_1x_2(x_1^2+x_2^2)}-1\le 0,\\ g_3\left( x\right)= & {} \frac{2.0}{x_1}-1\le 0,\\ g_4\left( x\right)= & {} \frac{x_1}{14}-1\le 0,\\ g_5\left( x\right)= & {} \frac{0.2}{x_2}-1\le 0,\\ g_6\left( x\right)= & {} \frac{x_2}{8}-1\le 0, \end{aligned}$$

Variable range:

$$\begin{aligned}{} & {} 2 \le x_1 \le 14,\\{} & {} 0.2 \le x_2 \le 0.8. \end{aligned}$$

The stress included in the column should be less than the buckling stress (constraint $g_1$) and the yield stress (constraint $g_2$). The mean diameter of the column is restricted between 2 and 14*cm* (constraint $g_3$ and $g_4$), and columns with thickness outside the range $0.2-0.8 cm$ are not commercially available (constraint $g_5$ and $g_6$). The mean diameter $d (x_1)$ and the thickness $t (x_2)$ vary in the range of [2,14] and [0.2,0.8].

This case was previously tackled by many scholars using various heuristic methods, including Social Network Search^49^, Cuckoo search algorithm^46^, krill herd algorithm^60^, Cooperation search algorithm^61^, and a Hybrid Generalized Reduced Gradient-Based Particle Swarm Optimizer^57^ respectively in the literatures.

**Figure 21:**
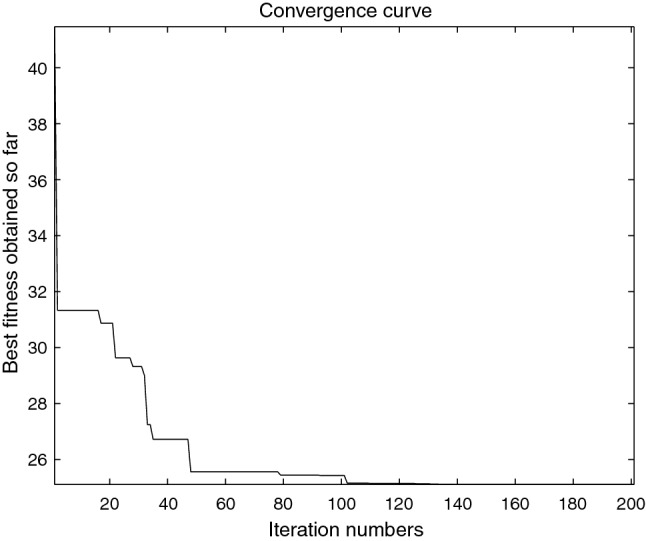
Convergence of tabular column.

The statistical results of LFGOA and other optimization methods are compared in Tables 27 and 28. Among the compared optimization algorithms, the LFGOA ranks first as superior to other approaches in optimizing the tubular column design, our method can find better geometric variables for this case. Hence, our result is feasible and verifies the effectiveness of the LFGOA algorithm. The results demonstrate that the LFGOA algorithm can provide reliable and very comprising solutions compared with the other algorithms.

**Table 27:** Results of LFGOA and competitive algorithms in solving the tubular column design.

Algorithm	Optimal values for variables		f(x)
	x1	x2	
LFGOA	2.5465	0.8000	**25.0574**
SNS	5.4512	0.2920	26.4995
HFBOA	5.4514	0.2920	26.5322
CS	5.4514	0.2920	26.5322
KH	5.4513	0.2920	26.5314
CSA	5.4512	0.2920	26.5314

The best of the comparison results are in [bold].

**Table 28:** Comparative results of LFGOA with other methods for tubular column.

Algorithm	Best	Mean	Std
LFGOA	**25.0574**	**25.4412**	0.2068
SNS	26.4995	26.4864	**0.0000**
CS	26.5322	26.5350	0.0019
KH	26.5314	26.5430	0.0180
CSA	26.5314	26.5316	0.0002
PSO_GRG	26.5313	26.5313	0.0000

The best of the comparison results are in [bold].

As shown in the Fig. 21, the convergence curve quickly converge towards the global optimum and the solutions was obtained instantly under satisfy all constraints.

## Results and discussion

As we can see in Section 5, seven real-world constrained engineering design examples including Himmelblau’s nonlinear optimization problem, Cantilever beam design, Car Side Impact Design, Gear train Design, Pressure vessel design, Speed Reducer Design, and tabular column design are selected to verify the proposed LFGOA algorithm. The LFGOA has been demonstrated to perform better than or be highly competitive with the other algorithms in the literature on the seven constrained engineering optimization problems, and can solve different real-world constrained engineering optimization problems. The advantages of LFGOA involve performing simply and having few parameters to regulate. The work here proves the LFGOA to be robust, powerful, and effective over all types of the other algorithms in the literature. Constrained engineering optimization evaluation is a good way for testing the performance of the metaheuristic algorithms, but it also has some limitations. For example, different tuning parameter values in the optimization methods might lead to significant differences in their performance. Also, constrained engineering optimization tests may arrive at fully different conclusions if the termination criterion changes. If we change the population size or the number of iterations, we might draw a different conclusion.

## Conclusion

This paper presented a novel enhancing Grasshopper Optimization Algorithm with Levy Flight algorithm, call LFGOA algorithm. Five metrics (i.e., search history, average fitness function, the best fitness history, the trajectory of the first dimension, and convergence curve) are implemented to investigate the LFGOA qualitatively. Next, 23 benchmark test functions to investigate the exploration, exploitation, local optima escape, and convergence performance of the LFGOA. The results demonstrated the effectiveness of LFGOA towards achieving optimal global solutions having more reliable convergence compared to other eight well-known optimization algorithms published in the literature. Freidman ranking test is applied to evaluate the efficacy of the LFGOA scientifically. The statistical results demonstrated that the LFGOA can guarantee the effectiveness of explorations while producing excellent exploitation, hence maintaining an equilibrium between exploitation and exploration strategies, which reveals the superior performance of the LFGOA in a statistical sense against other comparative algorithms. Moreover, seven real-world engineering problems are used to investigate the effectiveness of the LFGOA further. The results of the engineering design problems proved that the LFGOA achieved extremely better results against the other well-known optimization algorithms, and it can handle various constraints problems.

Of course, there are still many applications of the LFGOA algorithm worthy of further study because of the tremendous potential of the LFGOA algorithm. Moreover, the LFGOA algorithm can be used to solve constrained engineering optimization problems such as industry and engineering applications, and other application domains. There are several possible future directions and possible ideas worth investigating regarding the new variants of the LFGOA algorithm and its widespread applications, for example, features selection, job scheduling, and parameter optimization are still need to be resolved and can be suggested as future work.

## Data Availability

The datasets generated during or analysed during the current study are available from the corresponding author on reasonable request.
